# MAGIC population-based genetic dissection of yield-related traits under heat stress in wheat (*Triticum aestivum* L.)

**DOI:** 10.3389/fpls.2026.1790276

**Published:** 2026-04-07

**Authors:** Ananta Bag, Hari Krishna, P. N. Vinodh Kumar, Shiwani Meena, Narayana Bhat Devate, Rahul Meena, Sudhir Kumar, Ravindra Patil, Uday Govinda Reddy, Amit Kumar Singh, Badal Singh, Neelu Jain, Pradeep Kumar Singh, Gyanendra Pratap Singh

**Affiliations:** 1 Division of Genetics, Indian Agricultural Research Institute, New Delhi, India; 2 Division of Plant Physiology, Indian Agricultural Research Institute, New, Delhi, India; 3 Genetics and Plant Breeding Group, Agharkar Research Institute, Pune, India; 4 Department of Genetics and Plant Breeding, University of Agricultural Sciences, Dharwad, India; 5 Division of Genomic Resources, Indian Council of Agriculture Research (ICAR)-National Bureau of Plant Genetic Resources, New Delhi, India; 6 Division of Germplasm Evaluation, Indian Council of Agriculture Research (ICAR)-National Bureau of Plant Genetic Resources, New Delhi, India

**Keywords:** candidate genes, genome-wide association study (GWAS), heat susceptibility index (HSI), MAGIC population, marker trait association (MTA)

## Abstract

Heat stress poses a significant challenge to wheat productivity, necessitating the identification of genetic loci conferring yield stability and resilience. This study evaluated a multi-parent advanced generation intercross (MAGIC) population for biomass (BM), grain weight per spike (GWPS), thousand grain weight (TGW), yield per plot (YLD), and heat susceptibility indices (HSI_BM, HSI_GWPS, HSI_TGW, HSI_YLD) under timely-sown irrigated (TSIR) and late-sown irrigated (LSIR) conditions across Delhi, Dharwad, and Pune. The population exhibited substantial phenotypic variation and yield and biomass positively correlated and inversely related to heat susceptibility, highlighting potential for selection under stress conditions. Genome-wide association studies identified SNPs across nearly all wheat chromosomes associated with BM, GWPS, TGW, YLD and their corresponding heat susceptibility indices (HSIs), with phenotypic variance explained (PVE) ranging from 3-15%. Major-effect loci (e.g., AX-94529210, AX-95104040 and AX-95204353 for GWPS; AX-95210025 for TGW, AX-94496657 for BM; AX-94877518, AX-94942005 and AX-95118494 for YLD) and numerous minor-effect SNPs contributed to trait variation, reflecting a polygenic architecture. Allelic effect analysis demonstrated consistent enhancement of yield and reduction of heat susceptibility across environments. Collectively, this study underscores the MAGIC population as a valuable resource for dissecting complex traits and provides genomic insights for marker-assisted breeding of high-yielding, heat-tolerant wheat varieties.

## 1 Introduction

Wheat is one of the world’s most important staple crops and accounts for a major share of global trade (Borrill et al., 2015). Achieving stable and high wheat yields is therefore essential for sustaining global food security (Boyer and Westgate, 2004). Its production has increased from 69.9 million tons in 2012 to 800 million tons (2025) (https://www.fao.org/worldfoodsituation/csdb/en), the average annual growth rate of about 1% remains inadequate to reach the target of doubling yield by 2050 (Ray et al., 2013). Wheat yield is a complex quantitative trait influenced by numerous minor-effect genes. While significant progress has been made in developing genetic and genomic resources in recent decades, their limited integration into breeding programs has restricted their effective application for yield enhancement (Quraishi et al., 2017).Grain yield is determined by several yield-contributing components, such as grain number per spike, thousand-kernel weight, and biomass, all of them are quantitatively inherited (Zhang et al., 2010). In addition, stress and heat-related indices, including HSI_YLD (heat susceptibility index for yield), HSI_TGW (heat susceptibility index for thousand-kernel weight), HSI_GWPS (heat susceptibility index for grains weight per spike), and HSI_BM (heat susceptibility index for biomass), also play a significant role in determining yield stability under heat stress conditions.

Heat tolerance is a complex trait governed by multiple genes and characterized by intricate patterns of inheritance (Phuke et al., 2022; Saini et al., 2022; Bellundagi et al., 2022). Because it is controlled by many loci and strongly affected by genotype × environment interactions, heat tolerance generally exhibits low to moderate heritability (Kamara et al., 2021). Heat stress responses are highly complex because they depend on multiple interacting factors, including the crop species, the severity and duration of the temperature increase, and the developmental stage at which the stress occurs (Fahad et al., 2017; Prasad et al., 2008). Developing wheat genotypes with improved tolerance to elevated temperatures requires a clear understanding of the underlying physiological and molecular mechanisms, as well as plant performance under heat-stressed conditions (Cossani and Reynolds, 2012; Maity and Shrivastav, 2024). Research findings indicate that even a modest rise in temperature during critical reproductive stages can substantially impair wheat productivity. An increase of 1 °C around anthesis and grain filling has been associated with an approximate 6% decline in grain yield (Zhao et al., 2017; Mirosavljević et al., 2021) and about a 5% reduction in harvest index (Lawlor and Mitchell, 2000). Elevated temperatures during these stages also interfere with proper endosperm development, leading to fewer endosperm cells, smaller grain size, and lower thousand-kernel weight. Enhancing crop performance under abiotic stress conditions therefore requires the identification of reliable sources of stress tolerance and the subsequent introgression and deployment of favorable alleles into well-adapted, high-performing cultivars (Edae et al., 2014).

Genome-wide association studies (GWAS) and quantitative trait loci (QTL) mapping are the two principal approaches used to unravel the genetic architecture of complex traits (Risch and Merikangas, 1996). In wheat, conventional QTL mapping often provides low-resolution results that are restricted to the specific bi-parental population used. GWAS, on the other hand, offers a complementary approach with higher resolution and cost-effectiveness, enabling both gene discovery and molecular marker identification. While bi-parental populations are usually developed for targeted traits, GWAS utilizes diverse germplasm panels that are genotyped once and phenotyped for multiple traits, thereby maximizing the use of genetic variation within the population (Zhu et al., 2008). Genome-wide association studies (GWAS) leverage linkage disequilibrium (LD) within natural germplasm collections to detect genetic effects and have emerged as powerful tools in modern plant breeding (Alqudah et al., 2020). With advances in DNA marker technologies, particularly the development of high-density single nucleotide polymorphism (SNP) arrays, association mapping has increasingly enabled the identification of robust associations between genome-wide SNPs and traits of interest. Previously, highly significant marker trait associations (MTAs) (P < 0.001) for yield-related traits reported on chromosomes 1A, 1D, 2A, 3B, 4A, 4B, 5A, 5B, 5D, 6B, 7A, and 7B (Wang et al., 2017). Consistent marker trait associations (MTAs) for thousand grain weight (TGW) were identified on chromosomes 1B, 2B, 3A, 3B, 5A, 5B, 5D, 6B, and 7D (Zanke et al., 2015; Sehgal et al., 2019; Wang et al., 2021). In particular, a QTL hotspot on chromosome 5A (140–142 cM) was found to play a crucial role in controlling grain weight (Wang et al., 2021). The MTAs for grain weight per spike were detected on chromosome 6A, identifying a key genomic region for spike productivity and its potential use in marker-assisted selection for yield improvement (Sukumaran et al., 2015). Significant SNPs associated with biomass (BM) were identified at various growth stages on chromosomes 5A and 7A (Molero et al., 2019). A large proportion of previously reported QTL associated with yield and its component traits have been detected either under stress conditions or under optimal, non-stress environments (Saini et al., 2022; Su et al., 2018). However, only a limited number of investigations have evaluated whether these QTL remain stable and consistently expressed across contrasting environments, particularly under both heat stress and favorable conditions (Wang et al., 2021). In the context of climate variability, the identification of robust and stable QTL or favorable alleles that confer superior agronomic performance across diverse environments, including heat-prone settings, is crucial for sustainable wheat improvement. MAGIC population has emerged as a powerful tool for genetic mapping and breeding, offering several advantages over traditional biparental mapping populations and diverse germplasm used for GWAS. MAGIC populations combine multiple parental lines, which significantly increases the genetic diversity available for analysis. This diversity is crucial for capturing a wider array of alleles compared to biparental populations, which are typically limited to two parental lines (Mackay et al., 2014). Similarly, MAGIC populations undergo numerous rounds of intercrossing, leading to an increased rate of recombination compared to GWAS germplasm populations. Enhanced recombination in MAGIC population helps overcome linkage disequilibrium (LD) and produces shorter haplotypes, making the identification of QTLs more accurate and precise (Islam et al., 2016).

Despite the availability of genome-wide association studies (GWAS) and quantitative trait loci (QTL) mapping in wheat, limited studies have exploited multi-parent advanced generation inter-cross (MAGIC) populations for dissecting heat stress-related yield traits with high mapping resolution. Integrating high-density SNP genotyping with multi-parent populations may therefore enhance the power to detect stable loci controlling yield and heat tolerance. We hypothesize that MAGIC population-derived lines harbor substantial genetic variability and increased recombination, enabling high-resolution identification of genomic regions associated with yield components and heat susceptibility indices under stress conditions. The present study identified significant SNPs associated with key yield-related traits, including thousand grain weight (TGW), biomass (BM), grain weight per spike (GWPS), and heat susceptibility indices (HSI_YLD, HSI_TGW, HSI_GWPS, HSI_BM), through genome-wide association study (GWAS) using the wheat *35K Affymetrix SNP array* in a MAGIC population (Wang et al., 2022). The analyzed MAGIC population captured a wide range of genetic diversity, allowing high-resolution mapping of loci controlling both yield and stress-responsive traits. Significant MTAs were consistently detected across multiple chromosomes.

## 2 Materials and methods

### 2.1 Plant materials and growth conditions

In this study, a set of 248 MAGIC population-derived lines, along with their eight founder lines, were evaluated to assess yield-related traits and their heat susceptibility indices. The founder lines represent diverse wheat-growing regions and adaptation conditions across India: HD 3086 and HD 3043 from the North Western Plains Zone (NWPZ), adapted to timely sown irrigated and restricted irrigated conditions, respectively; HD 2985 and HI 1563 from the North Eastern Plains Zone (NEPZ), performing well under late sown irrigated conditions; HD 2932 and HI 1544 from the Central Zone, with adaptation to late sown and timely sown irrigated conditions, respectively; VL 907 from the Northern Hill Zone, suitable for late sown conditions under both irrigated and rainfed management; and GW 322 from the Peninsular Zone, adapted to timely sown irrigated conditions (**Table 1**). To reduce field variability and to ensure accurate phenotypic assessment, trials were laid out using an alpha-lattice design consisting of 16 blocks, with each block containing 16 plots.

**Table 1:** Pedigree and release information with target conditions of the eight founder lines used for MAGIC population development.

Founder line	Pedigree	Released zone	Condition
HD 3086	DBW14/HD2733//HUW468	NWPZ	TSIR
HD 3043	KAUZ/STAR//HD2643	NWPZ	TSRI
HD 2985	PJN/BOW//OPATA-85*2/3/CROC-1/TR.TA(224)//OPATA-85	NEPZ	LSIR
HD 2932	PBW 343/PASTOR	CZ	LSIR
HI 1544	HINDI 62/BOBWHITE/CPAN2099	CZ	TSIR
HI 1563	MACS2496*2/MC10	NEPZ	LSIR
VL 907	DYBR1982-83842ABVD50/VW9365//PBW343	NHZ	LSIR
GW 322	PBW173/GW196	PZ	TSIR

TSIR, timely-sown irrigated condition; LSIR, late-sown irrigated condition; NWPZ, North Western Plains Zone; NEPZ, North Eastern Plains Zone; CZ, Central Zone; NHZ, Northern Hills Zone; PZ, Peninsular Zone.

**Figure 1:**
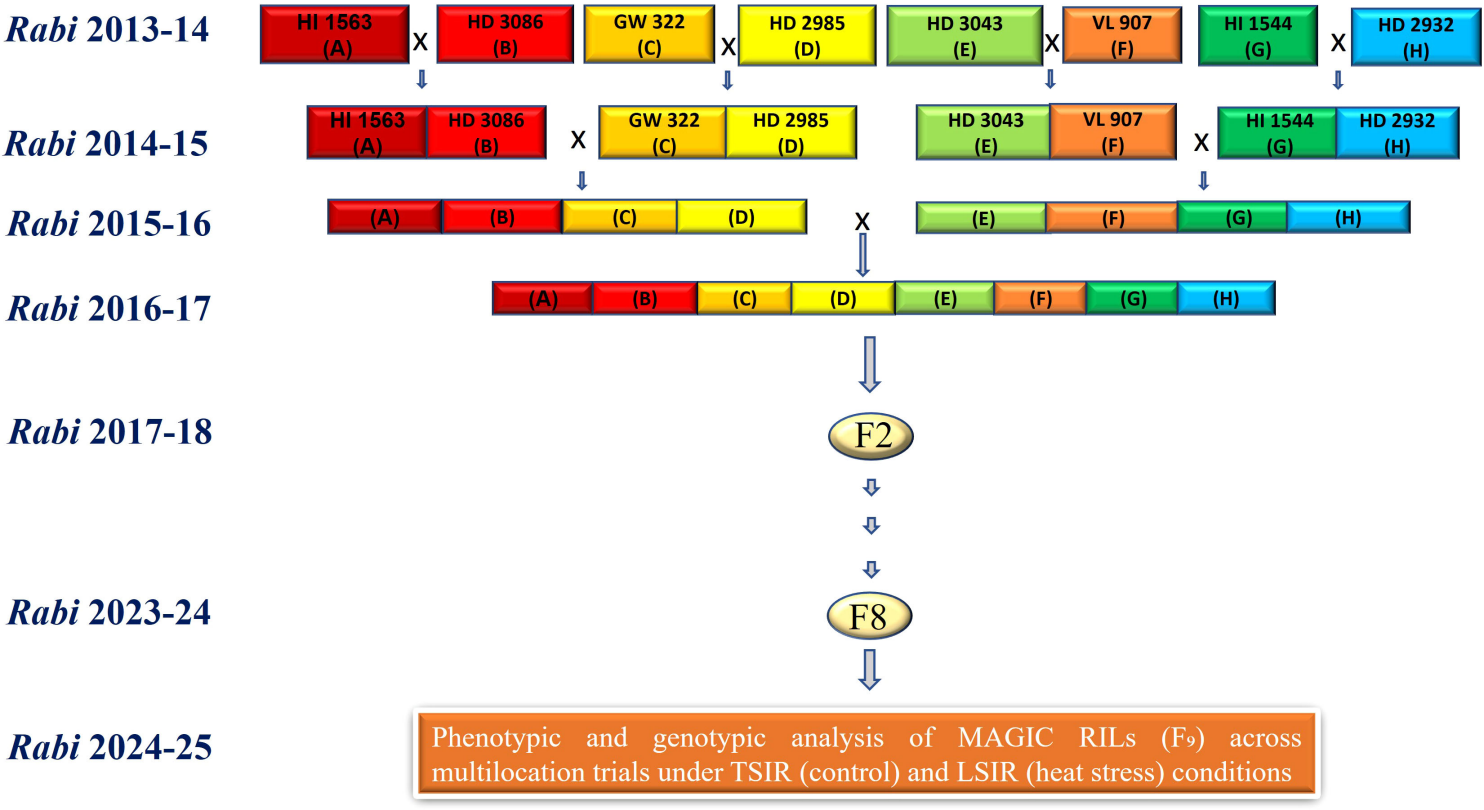
Timeline of development of the MAGIC population and its evaluation under heat stress.

The development of the MAGIC population was initiated during *Rabi* 2013–14 by implementing a half-diallel crossing scheme among the eight founder lines to generate 28 two-way crosses. These 28 two-way crosses were subsequently intercrossed to produce 72 four-way crosses during *Rabi* 2014-15. In the following season (*Rabi* 2015-16), 29 out of the 35 possible eight-way crosses were successfully developed. During *Rabi* 2016–17 these 8 way-crosses (F_1_) were grown to produce F_2_ seeds for next generation. By *Rabi* 2017-18, 4824 F_2_ seeds derived from these crosses were raised across 29 nodes, establishing the foundation of the population. The population was further advanced through the head-row method from the F_3_ to F_8_ generations between *Rabi* 2018–19 and 2023-24, thereby ensuring both genetic recombination and stabilization of lines (**Figure 1**).

At the onset of this study, the MAGIC population lines had reached the F_8_ generation, and bulked _9_ seeds were used for multi-environment evaluations. A total of 248 MAGIC-derived lines (_9_ RILs), along with their eight diverse founder lines representing different wheat-growing zones and adaptation conditions across India, were assessed across three locations IARI-Delhi, UAS-Dharwad, and ARI-Pune during 2024–25 growing season (one year) under both timely sown irrigated (TSIR) and late sown irrigated (LSIR) conditions for yield-related traits and heat susceptibility indices. The TSIR condition was considered the non-stress control, where the crop was sown at the recommended time under adequate irrigation. In contrast, the LSIR condition involved delayed sowing to expose the crop to elevated temperatures during the reproductive and grain-filling stages, thereby imposing terminal heat stress under irrigated conditions. Thus, late sowing served as the heat stress treatment while minimizing confounding moisture stress effects. Overall, the MAGIC population was evaluated across six distinct environments (three locations × two sowing conditions) for yield-related traits and heat susceptibility indices.

### 2.2 Heat susceptibility index

The heat susceptibility index (HSI) was calculated to evaluate heat tolerance using the method of Fischer and Maurer (1978):


$$HSI\;=\;\left(1\;-\;Y/Y_{P}\right)/D$$

Here, Y is the mean value of a trait for a genotype under heat stress, Y_P_ is the mean value under optimal conditions, and D represents the stress intensity, computed as 1 − X/X_P_, where X and X_P_ are the mean trait values of all genotypes under stress and optimal conditions, respectively. Based on HSI, genotypes were classified as highly tolerant (HSI ≤ 0.5), moderately tolerant (0.5 < HSI ≤ 1.0), or susceptible (HSI > 1.0) to heat stress (Khanna-Chopra and Viswanathan, 1999).

### 2.3 SNP genotyping

High-quality genomic DNA was extracted from young leaf tissues of 248 MAGIC population lines and parental genotypes using a modified CTAB method. DNA quality and integrity were assessed via agarose gel electrophoresis, and concentrations were quantified using a *NanoDrop spectrophotometer*. Only samples with concentrations above 25 ng/μl and minimal degradation were selected for genotyping to ensure reliable downstream analysis. Genotyping was performed using the *35K Axiom^®^ Wheat Breeder’s Array (Affymetrix)*. Allele calling and initial quality control were conducted using Axiom Analysis Suite software, adhering to the Axiom^®^ Best Practices Genotyping Workflow (https://documents.thermofisher.com/TFS-Assets/LSG/manuals/MAN0018363-AxiomDataAnalysis-UG-RUO.pdf.). After stringent quality filtering for missing data (<10%) and minor allele frequency (>5%), a total of 11,574 high-quality SNP markers were retained for analysis. These markers were well-distributed across all 21 wheat chromosomes, providing robust genome coverage for genome-wide association studies (GWAS), allelic effect analysis, and in silico functional annotation. To control the false positive rate in the GWAS analysis, the Bonferroni correction was applied. The genome-wide significance threshold was determined as –log_10_ (0.05/11574), which corresponded to a cut-off value of 5.36. Marker-trait associations exceeding this threshold were considered statistically highly significant. Genome-wide association analysis was performed using the BLINK (Bayesian-information and Linkage-disequilibrium Iteratively Nested Keyway) model implemented in GAPIT. BLINK was selected because it improves statistical power and computational efficiency while effectively controlling false positives compared to conventional MLM and FarmCPU models. The model iteratively incorporates significantly associated markers as fixed effects based on linkage disequilibrium patterns, thereby reducing confounding effects due to population structure and relatedness.

### 2.4 Statistical analysis

Phenotypic data were analyzed using R software. For each trait under TSIR and LSIR conditions across the three locations (Delhi, Dharwad, and Pune), descriptive parameters including mean, standard error (SE), standard deviation (SD), minimum, maximum, least significant difference (LSD), coefficient of variation (CV), mean sum of squares (MSS), significance, and broad-sense heritability (h²) were computed using ‘‘*agricolae’’* package in R software. Best Linear Unbiased Predictors (BLUPs) and adjusted means were estimated using Meta-R software (Alvarado et al., 2015) by fitting models that accounted for replication, block, genotype, and genotype-by-environment (G×E) interactions. In these analyses, genotypes and covariates were considered fixed effects for BLUEs and random effects for BLUPs, thereby improving the accuracy of genotype performance estimates across multi-environment trials (METs). Additionally, pairwise Pearson correlation coefficients (r) were calculated to evaluate linear associations among traits, and correlation matrices were visualized using the “*corrplot*” package in R. Principal component analysis (PCA) was also performed using ‘‘*FactoMineR’’* and *‘‘factoextra’’* in R.

### 2.5 Population structure and linkage disequilibrium analysis

Population structure of the wheat germplasm subset was inferred using STRUCTURE (Pritchard et al., 2010), with a burn-in period of 100,000 iterations followed by 100,000 Markov Chain Monte Carlo (MCMC) replications. The optimal number of subpopulations was determined using the ΔK method proposed by Evanno et al., 2005 as implemented in *STRUCTURE Harvester*. Principal Component Analysis (PCA) was conducted using 11,574 SNP markers to validate the clustering patterns. Both computation and graphical visualization were performed using the GAPIT package in R (Lipka et al., 2012). In addition, a Neighbor-Joining (NJ) tree was constructed, and the ΔK values were calculated to further confirm the population stratification.

Linkage disequilibrium (LD) was estimated by calculating pairwise squared allele frequency correlations (r²) between adjacent markers across the genome using TASSEL (Bradbury et al., 2007). Genome-wide LD decay was assessed by fitting a nonlinear regression model based on the modified Hill and Weir method (Hill and Weir, 1988) and the LD decay pattern was visualized using R software.

### 2.6 Genome wide association study

Genome-wide association analysis was carried out in R using GAPIT version 3.0, applying the BLINK model (Bayesian-information and Linkage-disequilibrium Iteratively Nested Keyway; Huang et al. (2019) to identify quantitative trait nucleotides (QTNs) while controlling for population structure and linkage disequilibrium to reduce false positives. High-quality filtered SNP markers along with BLUP values derived across locations and sowing conditions were used as input for the analysis, following the GAPIT user guidelines (https://zzlab.net/GAPIT/gapit_help_document.pdf). Model performance was evaluated using quantile-quantile (Q-Q) plots, where alignment along the diagonal indicated an adequate fit, and deviations at the tail highlighted significant associations. Significant marker–trait associations were identified at a threshold of -log_10_(p) > 3 (p < 0.001), and the results were visualized using Manhattan plots to depict the genomic locations and significance levels of associated SNPs.

### 2.7 Allelic effect analysis of significant SNPs

Allelic effect analysis was performed to assess the functional contribution of significant SNPs associated with grain yield per plot (YLD), biomass (BM), thousand grain weight (TGW), grain weight per spike (GWPS), and their corresponding heat susceptibility indices (HSI_YLD, HSI_BM, HSI_TGW, HSI_GWPS) in the MAGIC population. For each SNP identified through GWAS, the population was divided according to allelic classes, and mean trait values were compared to estimate the phenotypic effects of favorable versus unfavorable alleles. Standard deviation was calculated, and t-tests were conducted to evaluate the significance of differences between allelic classes. BLUP-adjusted trait values across environments were employed to ensure robust estimation of allelic effects under both timely sown irrigated (TSIR) and late sown irrigated (LSIR) conditions.

### 2.8 *In-silico* candidate gene analysis

Potential candidate genes linked to trait-associated SNPs were identified by performing BLAST searches with the sequences of significant markers on the *Ensembl Plants platform* (https://plants.ensembl.org/Triticum_aestivum/Tools/Blast) against the bread wheat reference genome *IWGSC RefSeq v1.0* (Yates et al., 2022). This enabled accurate localization of SNPs within the genome. To capture genes within the typical range of linkage disequilibrium in wheat, genomic regions spanning ±500 kb around each associated SNP were examined using the “*Region Comparison*” tool in *Ensembl Plants*, and putative candidate genes along with their transcript IDs were retrieved. Functional annotation of the identified genes was conducted using *UniProt*, providing information on the encoded proteins and their biological roles.

## 3 Results

### 3.1 Phenotypic evaluation and identification of productive MAGIC lines through MGIDI analysis using BLUP-based multi-location estimates

Significant variation was observed for all studied traits across locations and conditions. The mean biomass (BM) ranged from 0.76 kg plot ^-1^(LSIR, Delhi) to 1.50 kg plot ^-1^ (TSIR. Pune), while grain weight per spike (GWPS) varied from 1.43 g (LSIR, Pune) to 2.34 g (TSIR, Delhi). Thousand grain weight (TGW) exhibited a wide range from 36.3 g (LSIR, Dharwad) to 51.5 g (TSIR, Dharwad), and grain yield (YLD) from 0.23 kg plot ^-1^ (LSIR, Delhi) to kg plot ^-1^ (TSIR, Pune). The Heat Susceptibility Index (HSI) values ranged from 0.97 to 1.04 across traits and sites, with heritability of 0.53-0.75, indicating stable genetic performance under heat stress (**Table 2**). Combined analysis of variance revealed highly significant (P ≤ 0.001) effects of genotype (G), environment (E), and genotype × environment (G × E) interaction for all major yield traits including biomass (BM), grain yield (YLD), grain weight per spike (GWPS), and thousand grain weight (TGW). The environmental effect showed the largest mean squares across traits, particularly for TGW and BM, indicating strong environmental influence on trait expression. Significant G × E interactions further suggest differential genotypic responses across environments, highlighting the importance of multi-environment evaluation. For heat susceptibility indices (HSI-BM, HSI-YLD, HSI-GWPS, and HSI-TGW), genotypic effects were highly significant, demonstrating substantial genetic variability for heat tolerance within the MAGIC population. The G × E interaction was also significant for all HSI traits, indicating variability in heat stress response across environments (**Table 3**). Overall, the results confirm the presence of ample genetic variability and significant genotype × environment interaction for yield and heat tolerance–related traits in the MAGIC population.

**Table 2:** Descriptive statistics of productivity traits and heat susceptibility indices under different sowing conditions and environments.

Traits	Conditions	Mean	SE	SD	Min	Max	h^2^	LSD	CV
BM	LS_DL	0.76	0.01	0.24	0.13	1.45	0.67	0.2	13.5
BM	TS_DL	1.28	0.01	0.2	0.82	1.85	0.69	0.13	5.18
BM	LS_DHAR	0.79	0.01	0.23	0.158	1.37	0.65	0.17	11.4
BM	TS_DHAR	1.03	0.01	0.24	0.19	1.79	0.7	0.23	12
BM	LS_PUNE	1.18	0.01	0.18	0.762	1.62	0.63	0.12	5.29
BM	TS_PUNE	1.5	0.01	0.18	1	1.92	0.68	0.13	4.35
GWPS	LS_DL	1.87	0.02	0.37	0.86	2.75	0.75	0.31	8.51
GWPS	TS_DL	2.34	0.02	0.43	1.02	3.46	0.78	0.37	8.26
GWPS	LS_DHAR	1.6	0.01	0.32	0.82	2.45	0.71	0.28	9.17
GWPS	TS_DHAR	2.12	0.01	0.25	1.49	2.79	0.74	0.28	8.06
GWPS	LS_PUNE	1.43	0.01	0.32	0.573	2.29	0.7	0.2	7.13
GWPS	TS_PUNE	1.9	0.01	0.27	1.02	2.62	0.76	0.2	5.51
TGW	LS_DL	39	0.2	4.52	26.7	50.6	0.78	3.83	5.25
TGW	TS_DL	48.9	0.23	5.2	31.3	61.1	0.81	3.92	4.12
TGW	LS_DHAR	36.3	0.2	4.53	23.19	49.1	0.76	3.7	5.31
TGW	TS_DHAR	51.5	0.27	6.07	34.26	66.4	0.79	4.29	4.37
TGW	LS_PUNE	36.8	0.2	4.55	23.7	48.2	0.78	2.07	2.77
TGW	TS_PUNE	43.2	0.15	3.38	34.32	52.8	0.82	2.51	2.99
YLD	LS_DL	0.23	0	0.08	0.06	0.46	0.62	0.08	19.1
YLD	TS_DL	0.4	0	0.09	0.15	0.66	0.67	0.11	16.2
YLD	LS_DHAR	0.24	0	0.07	0.035	0.43	0.65	0.08	18.3
YLD	TS_DHAR	0.35	0	0.09	0.063	0.57	0.67	0.09	15.5
YLD	LS_PUNE	0.4	0	0.07	0.167	0.59	0.61	0.05	5.95
YLD	TS_PUNE	0.48	0	0.06	0.31	0.65	0.66	0.05	4.88
HSI_BM	DL	0.98	0.02	0.45	0.024	2.16	0.65	0.41	22.2
HSI_BM	DHAR	1.01	0.03	0.76	0.01	2.98	0.68	0.79	44
HSI_BM	PUNE	0.99	0.02	0.49	0.009	2.41	0.66	0.47	25.9
HSI_GWPS	DL	0.97	0.03	0.62	0.011	2.75	0.75	0.67	40
HSI_GWPS	DHAR	1	0.02	0.55	0.021	2.49	0.69	0.61	37.1
HSI_GWPS	PUNE	1.04	0.03	0.66	0.004	2.9	0.72	0.49	24.4
HSI_TGW	DL	0.99	0.02	0.38	0.015	2.35	0.75	0.42	25.1
HSI_TGW	DHAR	0.98	0.01	0.33	0.008	2.04	0.73	0.31	17.2
HSI_TGW	PUNE	1	0.03	0.61	0.004	3.1	0.78	0.44	22.3
HSI_YLD	DL	0.99	0.02	0.41	0.071	1.9	0.63	0.46	29.6
HSI_YLD	DHAR	0.99	0.02	0.52	0.024	2.19	0.53	0.57	40
HSI_YLD	PUNE	1	0.03	0.69	0.002	3.25	0.65	0.59	31.5

LS, Late sown irrigated condition (LSIR), TS, Timely sown irrigated condition (TSIR); DL, Delhi, DHAR, Dharwad, PUNE, Pune; h², Broad-sense heritability, SE, Standard Error, SD, Standard Deviation, CV, Coefficient of Variation, LSD, Least Significant Difference.

**Table 3:** Combined analysis of variance (ANOVA) for yield-related traits and heat susceptibility indices in MAGIC population across multiple environments.

Traits	Sources	Df	SS	MSS	F value	Sign
BM	Gen	255	34.45	0.140	17.230	***
	Env	5	208.80	41.760	5326.430	***
	Rep	1	0.043	0.040	5.450	*
	Gen: Env	1275	89.38	0.070	8.940	***
	Env: Rep : Block	185	2.74	0.015	1.880	***
	Residuals	1350	10.58	0.010		
YLD	Gen	255	5.481	0.021	10.768	***
	Env	5	24.764	4.952	2481.308	***
	Rep	1	0.004	0.004	2.214	**
	Gen: Env	1275	10.216	0.008	4.014	***
	Env: Rep : Block	185	0.776	0.004	2.103	***
	Residuals	1350	2.695	0.002		
GWPS	Gen	255	108.909	0.427	18.718	***
	Env	5	280.838	56.168	2461.564	***
	Rep	1	0.363	0.363	15.596	***
	Gen: Env	1275	187.310	0.147	6.438	***
	Env: Rep : Block	185	9.149	0.049	2.167	***
	Residuals	1350	30.804	0.023		
TGW	Gen	255	28511	111.800	33.6827	***
	Env	5	104818	20964	6315.298	***
	Rep	1	36	35.900	10.826	**
	Gen: Env	1275	35666	28	8.427	***
	Env: Rep : Block	185	1331	7.200	2.167	***
	Residuals	1350	4481	3.300		
HSI_BM	Gen	255	144.410	0.566	5.492	***
	Env	2	0.211	0.105	1.022	
	Rep	1	2.338	2.338	22.678	***
	Gen: Env	510	282.129	0.553	5.365	***
	Env: Rep : Block	92	20.312	0.220	2.141	***
	Residuals	675	69.597	0.103		
HSI_YLD	Gen	255	136.242	0.534	4.731	***
	Env	2	0.049	0.024	0.215	
	Rep	1	2.706	2.706	23.965	***
	Gen: Env	510	228.362	0.448	3.965	***
	Env: Rep : Block	92	22.819	0.248	2.196	***
	Residuals	675	76.231	0.113		
HSI_GWPS	Gen	255	179.537	0.704	5.937	***
	Env	2	1.240	0.620	5.225	**
	Rep	1	1.128	1.128	9.507	**
	Gen: Env	510	294.892	0.578	4.875	***
	Env: Rep : Block	92	15.502	0.169	1.421	**
	Residuals	675	80.055	0.119		
HSI_TGW	Gen	255	112.809	0.442	9.345	***
	Env	2	0.169	0.084	1.786	
	Rep	1	1.050	1.050	22.191	***
	Gen: Env	510	163.098	0.320	6.755	***
	Env: Rep : Block	92	9.765	0.106	2.242	***
	Residuals	675	31.954	0.047		

Gen, Genotype, Rep, Replication, Env, Environment, Df, Degree of FreedomSS, Sum of Squares, MSS, Mean Sum of Squares; Significance: *** *p* < 0.001, ** *p* < 0.01, * *p* < 0.

Based on the Multitrait genotype-ideotype distance index (MGIDI) computed from BLUP values of productive traits across locations, productive MAGIC lines- *MAGIC-196*, *MAGIC-165*, *MAGIC-15*, *MAGIC-30*, *MAGIC-49*, *MAGIC-71*, and *MAGIC-145* were identified as superior performers. These lines exhibited the lowest MGIDI values, indicating their proximity to the ideotype and balanced performance across multiple yield-related traits. The consistently low index values suggest that these genotypes combined favorable alleles for key productive attributes such as grain yield, biomass accumulation.

### 3.2 Correlation

**Figure 2:**
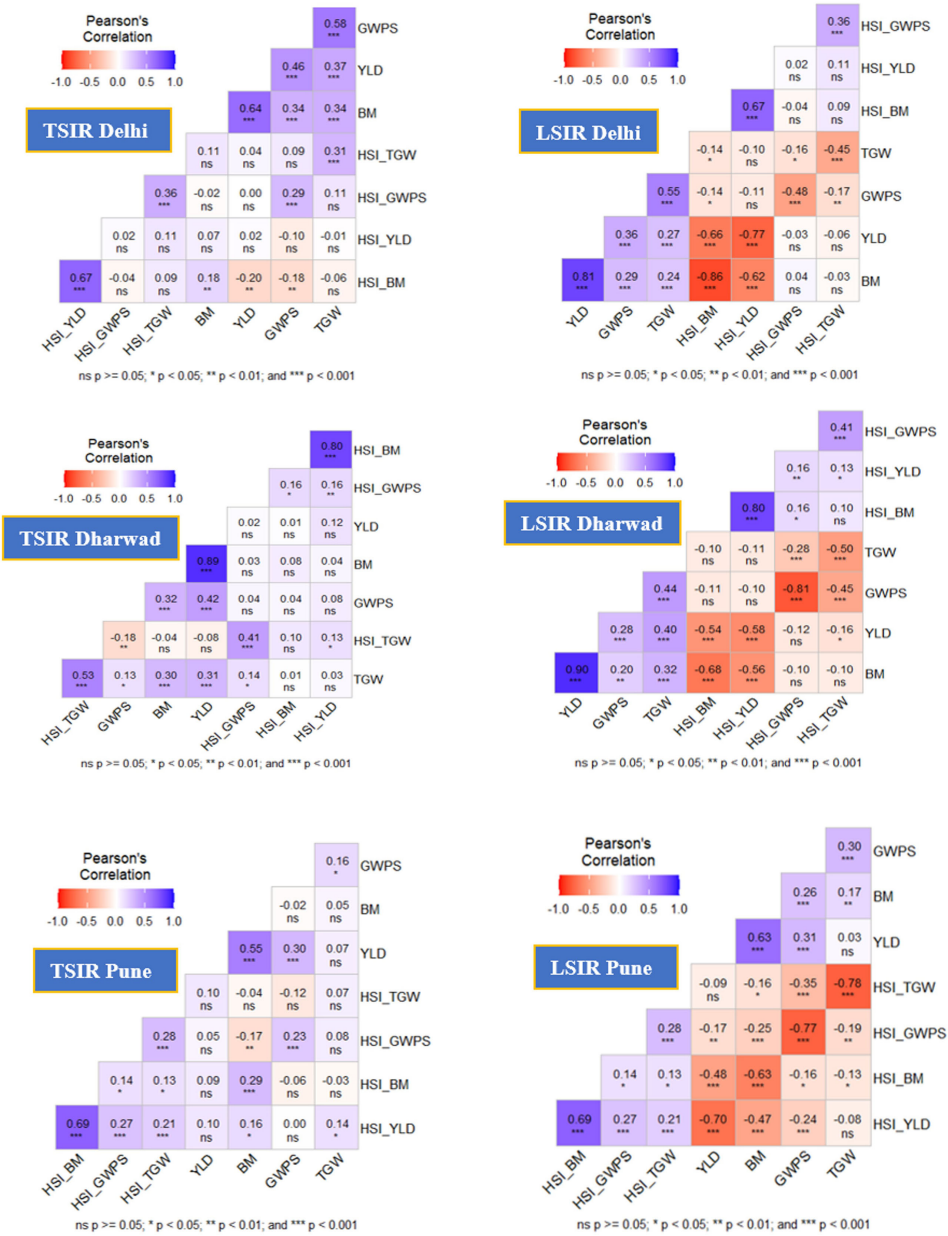
Correlation among the studied traits evaluated across three locations: IARI-Delhi; UAS-Dharwad; and ARI-Pune under TSIR and LSIR conditions.

Correlation analysis in the MAGIC population revealed that under TSIR conditions, biomass (BM) was consistently positively correlated with grain yield (YLD) across all locations Delhi (*r*, 0.64), Dharwad (*r*, 0.89), and Pune (*r*, 0.55) and showed significant associations with grain weight per spike (GWPS) and thousand-grain weight (TGW). YLD also displayed moderate positive correlations with GWPS and TGW, while GWPS and TGW were moderately correlated. Heat susceptibility indices (HSIs) generally showed weak or non-significant correlations under TSIR, except for strong positive associations between HSI_BM and HSI_YLD at Dharwad (*r*, 0.80) and Pune (*r*, 0.69). Under LSIR conditions, YLD exhibited very strong positive correlations with BM at all locations (Delhi *r*, 0.81, Dharwad *r*, 0.90, Pune *r*, 0.63) and moderate positive associations with GWPS and TGW (**Figure 2**). Strong negative correlations were observed between yield-related traits and their respective HSIs, including YLD vs. HSI_YLD, BM vs. HSI_BM, GWPS vs. HSI_GWPS, and TGW vs. HSI_TGW.

### 3.3 Principal component analysis

**Figure 3:**
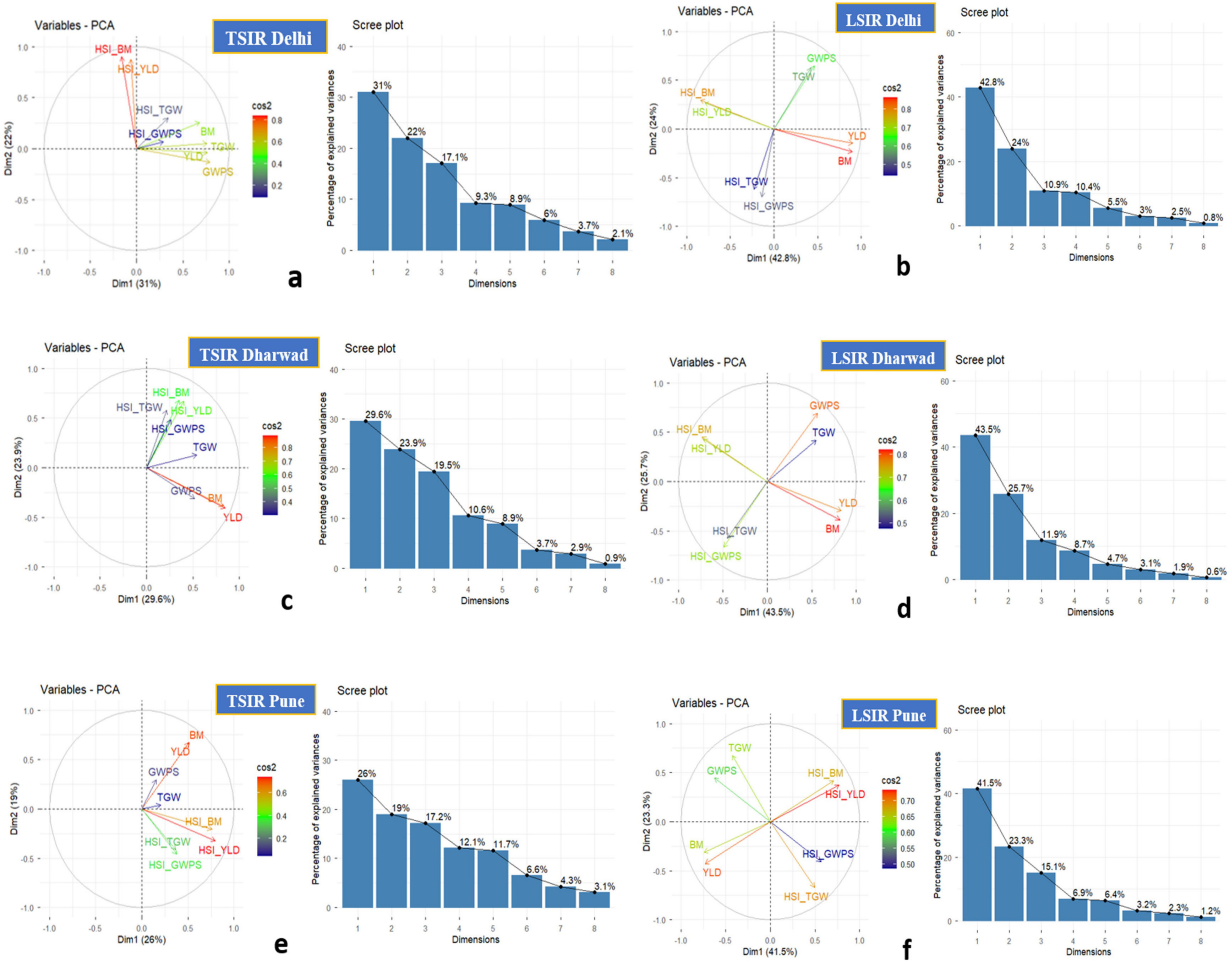
PCA plots for various conditions at different locations. **(a)** Delhi TSIR, **(b)** Delhi LSIR, **(c)** Dharwad TSIR, **(d)** Dharwad LSIR, **(e)** Pune TSIR, **(f)** Pune LSIR.

Principal component analysis (PCA) of the MAGIC population under different sowing conditions and locations revealed a consistent separation between productivity and heat susceptibility traits. Under TSIR conditions, the first two components explained 45-53% of variance, with yield-related traits (YLD, BM, GWPS, TGW) clustering together and HSIs (HSI_BM, HSI_YLD, HSI_GWPS, HSI_TGW) forming separate clusters, indicating negative correlations between productivity and heat susceptibility. Under LSIR, Dim1 and Dim2 captured 64-69% of variance, with YLD and BM strongly negatively correlated with HSI_BM and HSI_YLD, while GWPS and TGW showed moderate orthogonality, reflecting weaker associations with overall productivity (**Figures 3a-f**).

### 3.4 Population structure and genome-wide linkage disequilibrium in the MAGIC population

**Figure 4:**
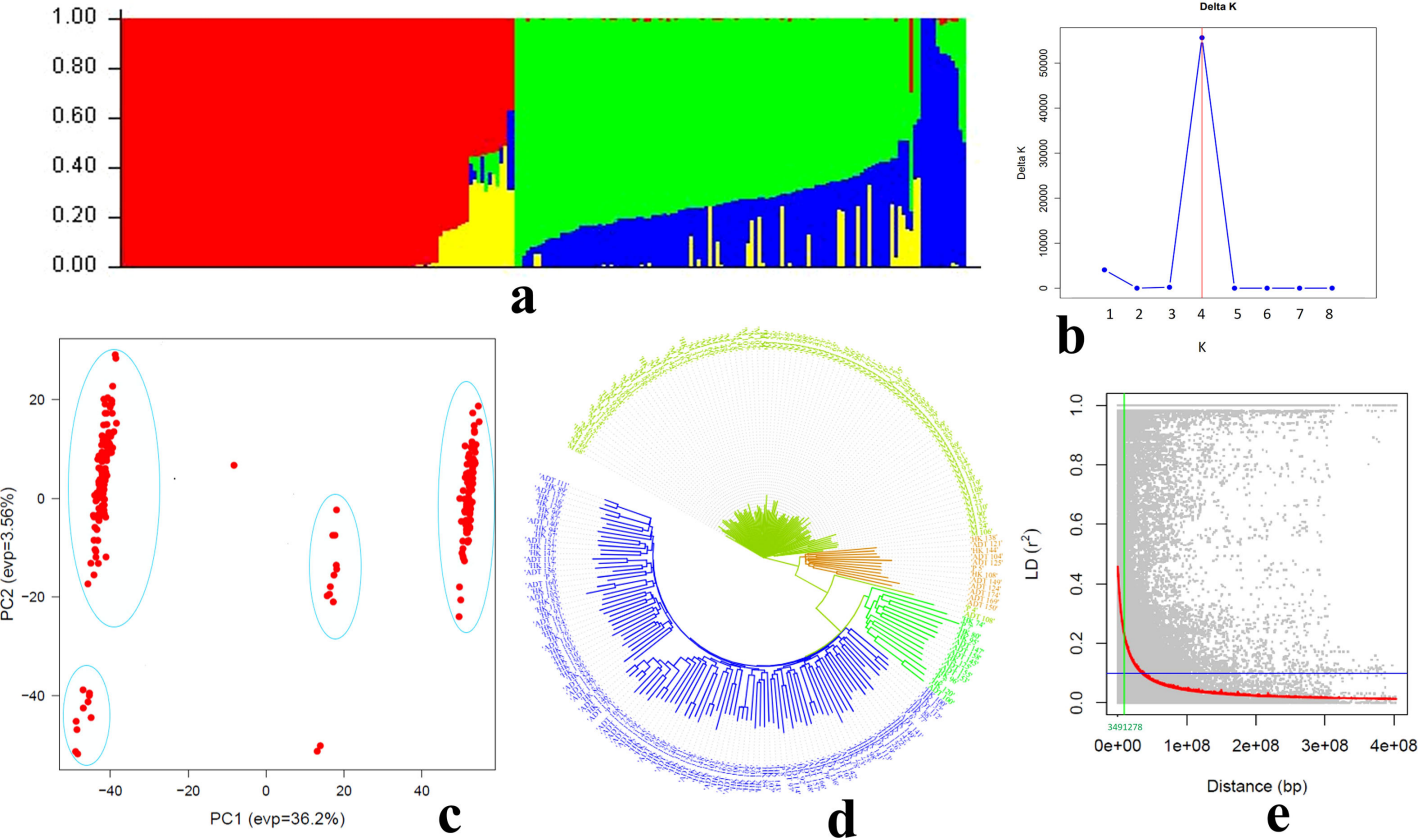
Population structure, genetic relationships and linkage disequilibrium analysis of the MAGIC population. **(a)** Population structure; **(b)** ΔK method for determining the optimal number of subpopulations; **(c)** principal component analysis (PCA) showing genetic differentiation among lines; **(d)** neighbor-joining phylogenetic tree illustrating genetic relationships and **(e)** genome-wide linkage disequilibrium (LD) decay plotted as r² against physical distance.

Population structure analysis using STRUCTURE revealed the presence of four distinct subpopulations (K, 4) within the wheat MAGIC population, as indicated by the highest ΔK value obtained through STRUCTURE Harvester. The clustering pattern was further supported by Principal Component Analysis (PCA) performed using 11,574 SNP markers in GAPIT, as well as by the Neighbor-Joining phylogenetic tree constructed using TASSEL. Genome-wide linkage disequilibrium (LD) analysis showed an average LD block size of 3.49 Mb across the whole genome (**Figure 4**).

### 3.5 Marker trait associations

**Figure 5:**
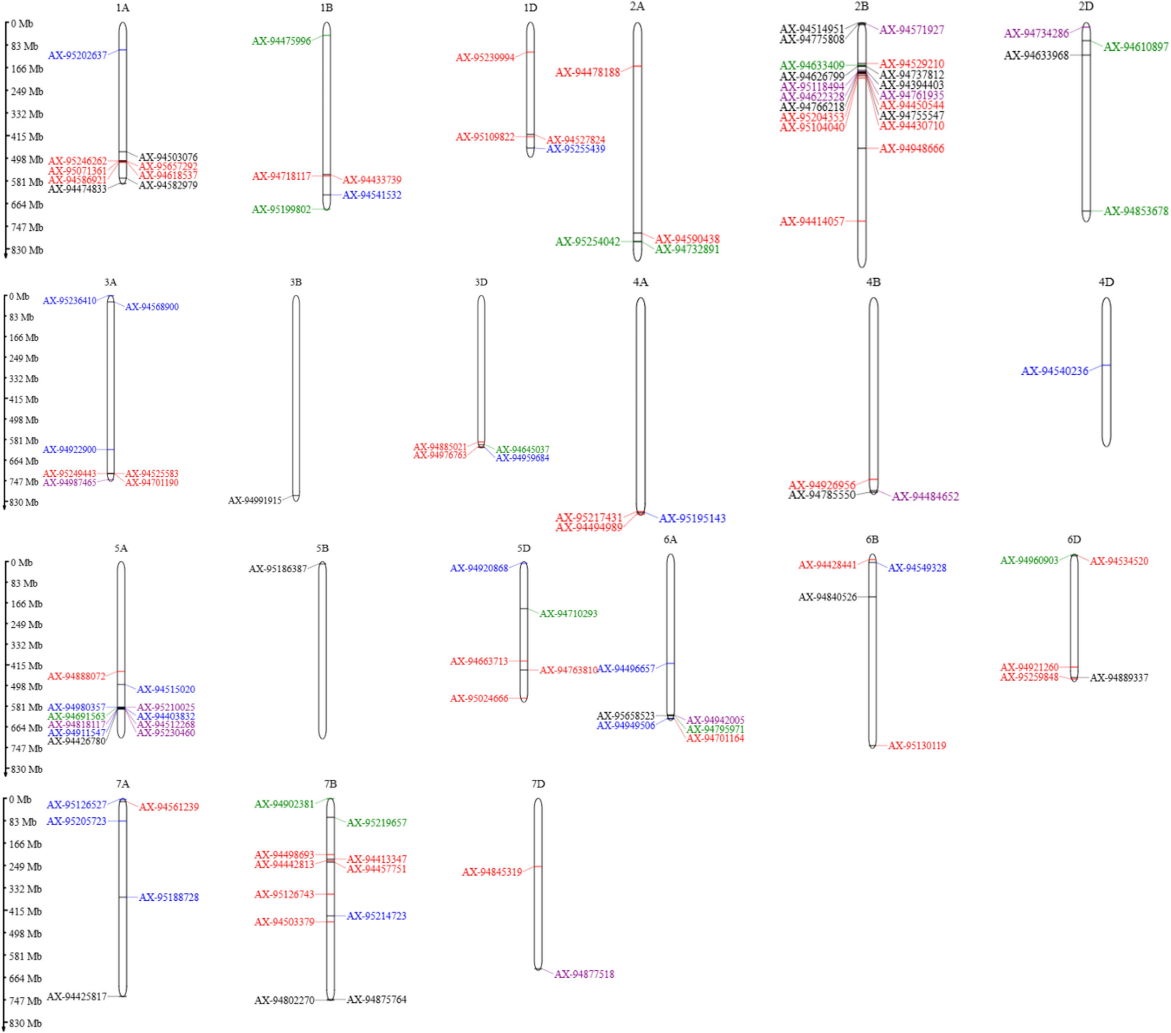
Chromosomal distribution of significant SNPs associated with productivity and heat susceptibility traits in the MAGIC wheat population. This illustrates the physical positions of significant SNPs identified through GWAS across the 21 wheat chromosomes (A, B, and D genomes). SNPs associated with biomass (BM) and HSI_BM are shown in blue, grain weight per spike (GWPS) and HSI_GWPS in red, thousand grain weight (TGW) and HSI_TGW in green, and grain yield per plot (YLD) and HSI_YLD in black. Pleiotropic SNPs, associated with multiple traits, are highlighted in purple. The distribution reveals key genomic regions harboring loci influencing yield and heat tolerance under different sowing conditions.

Following the GWAS analysis, significant marker-trait associations (MTA) were identified using genome-wide SNP markers. To minimize false positives and ensure stringent selection, a filtering criterion of LOD score > 3 was applied. This rigorous approach led to the identification of 114 MTAs, representing a substantial number of reliable associations that can be effectively utilized for marker-assisted selection (MAS) and genomic selection (GS) in wheat breeding. Detailed information on these MTAs, including their p-values and R² values, is provided in **Table 4** and **Supplementary Table 1**, while the significant SNPs are illustrated using Manhattan plots (**Supplementary Figure 1**-**3**) and their chromosome-wise distribution is depicted in **Figure 5**.

**Table 4:** Significant marker-trait associations identified across environments for BM, GWPS, TGW, YLD, and their corresponding Heat-stress indices in the MAGIC population.

Traits	Environment	SNP	Chr. No.	Pos. in Mb	P value	R^2^	-log(p)
BM	TS_DHAR	AX-94942005	6A	598.18	3.00E-09	9.25	8.49
BM	TS_DHAR	AX-95210025	5A	585.41	1.00E-06	7.83	5.83
BM	TS_DHAR	AX-94496657	6A	404.7	3.00E-06	13.7	5.48
BM	LS_PUNE	AX-94818117	5A	591.46	1.00E-05	7.68	4.98
BM	TS_PUNE	AX-94541532	1B	632.53	2.00E-05	5.6	4.67
BM	LS_PUNE	AX-94920868	5D	8.0338	4.00E-05	6.32	4.37
BM	LS_PUNE	AX-95210025	5A	585.41	5.00E-05	5.66	4.32
BM	TS_PUNE	AX-95205723	7A	85.836	1.00E-04	5.45	4.01
GWPS	TS_PUNE	AX-94428441	6B	20.999	4.00E-07	7.02	6.43
GWPS	TS_DL	AX-94529210	2B	135	3.00E-05	15.3	4.6
GWPS	TS_DL	AX-94948666	2B	412.66	4.00E-05	9.53	4.41
GWPS	LS_DL	AX-95204353	2B	168.61	7.00E-05	7.78	4.18
GWPS	TS_DL	AX-95204353	2B	168.61	7.00E-05	11	4.18
GWPS	TS_DL	AX-95118494	2B	161.15	7.00E-05	10	4.18
GWPS	TS_DL	AX-94430710	2B	172.98	7.00E-05	10.1	4.17
GWPS	TS_DL	AX-94622328	2B	162.93	8.00E-05	9.91	4.09
TGW	TS_DHAR	AX-95210025	5A	585.41	3.00E-13	13.3	12.5
TGW	TS_DHAR	AX-94960903	6D	4.6986	0.0001	5.72	3.94
TGW	LS_DHAR	AX-94691563	5A	585.49	0.0001	4.44	3.86
YLD	TS_DHAR	AX-94942005	6A	598.18	4.00E-12	11.5	11.4
YLD	TS_DHAR	AX-95210025	5A	585.41	3.00E-07	8.08	6.48
YLD	TS_DHAR	AX-94737812	2B	141.85	4.00E-05	8.02	4.4
YLD	TS_DHAR	AX-94626799	2B	141.88	4.00E-05	8.02	4.4
YLD	LS_DHAR	AX-94426780	5A	595.54	5.00E-05	6.77	4.29
YLD	LS_DL	AX-95118494	2B	161.15	6.00E-05	10.6	4.25
HSIBM	DL	AX-94987465	3A	744.06	0.0001	4.94	3.88
HSI_BM	PUNE	AX-94922900	3A	620.76	0.0002	4.53	3.71
HSI_GWPS	DHAR	AX-94976763	3D	609.94	4.00E-06	3.42	5.35
HSI_GWPS	DHAR	AX-95239994	1D	109.98	6.00E-06	5.32	5.23
HSI_GWPS	DHAR	AX-95249443	3A	719.3	5.00E-05	6.42	4.33
HSI_GWPS	DL	AX-95657292	1A	508.32	6.00E-05	3.75	4.25
HSI_TGW	PUNE	AX-94734286	2D	16.358	5.00E-05	10.7	4.32
HSI_TGW	DHAR	AX-94475996	1B	48.467	0.0002	4.22	3.66
HSI_YLD	PUNE	AX-95186387	5B	10.169	0.0001	3.91	3.91
HSI_YLD	PUNE	AX-94875764	7B	746.52	0.0002	5	3.78

GWPS, rain weight per spike; TGW, Thousand-grain weight; BM, Biomass; YLD,Grain yield; HSI_GWPS, HSI_TGW, HSI_BM, HSI_YLD , Heat susceptibility indices for respective traits; TS,– Timely sown; LS, Late Sown Irrigated (LSIR); TS, Timely Sown Irrigated (TSIR); DL, Delhi; DHAR, Dharwad; PUNE, Pune.

Among the evaluated traits, the highest number of MTAs was detected for BM (18), GWPS (15), TGW (10), and YLD (24). For the heat susceptibility indices, 12 MTAs were identified for HSI_BM, 33 for HSI_GWPS, 7 for HSI_TGW, and 9 for HSI_YLD. In addition, 13 pleiotropic SNPs were identified, each significantly associated with multiple traits, suggesting shared genetic control and highlighting potential genomic regions for simultaneous improvement of correlated traits. Several major-effect loci were consistently detected across environments, including AX-94529210, AX-95104040 and AX-95204353 for grain weight per spike (GWPS), AX-95210025 for thousand grain weight (TGW), AX-94496657 for biomass (BM), and AX-94877518, AX-94942005 and AX-95118494 for grain yield (YLD).

### 3.6 Identification of putative candidate genes and allelic effect of associated SNPs

The identified SNPs for biomass (BM), grain weight per spike (GWPS), thousand grain weight (TGW), yield (YLD), and their respective heat stress indices were linked to genes involved in protein phosphorylation and kinase-mediated signaling, ubiquitin-mediated protein turnover, transcriptional regulation (WRKY, MYB, AT-hook), redox and metabolic processes (cytochrome P450, Fe²^+^/2-oxoglutarate–dependent dioxygenases), calcium-mediated signaling (EF-hand proteins), and stress-adaptive developmental pathways (NB-ARC and AAA+ ATPase proteins) (**Supplementary Table 3**). On chromosome 5A, SNP AX-95210025 (585.41 Mb) was located near *TraesCS5A02G388800* encoding a serine/threonine protein kinase, while AX-94818117 (591.46 Mb) was positioned close to *TraesCS5A02G396800*, a WRKY transcription factor, both implicated in biomass regulation under stress. On chromosome 2B, AX-94948666 (412.66 Mb) was associated with *TraesCS2B02G295900* (RING-type E3 ubiquitin transferase) and AX-95118494 (161.15 Mb) with *TraesCS2B02G186100* (Cytochrome P450), linked to grain weight regulation under heat stress. SNP AX-94949506 (610.95 Mb) on 6A co-localized with *TraesCS6A02G404300/400*, encoding a MYB/SANT-like protein involved in stress-responsive biomass control. Additionally, AX-95249443 on 3A was associated with EF-hand domain-containing proteins (*TraesCS3A02G492200/400/500/600*) related to calcium-mediated heat signaling. SNP AX-94734286 (16.36 Mb) on 2D was located near *TraesCS2D02G045100*, encoding xyloglucan endotransglucosylase/hydrolase, implicated in TGW regulation under heat stress.

Allelic effect analysis in the MAGIC population revealed that multiple SNPs significantly influenced productivity traits and their heat susceptibility indices (HSIs) across Delhi, Dharwad, and Pune under both timely and late-sown irrigated conditions. For GWPS, the T allele of AX-94529210 at Delhi (+0.39 g) and the C allele of AX-95204353 under late-sown conditions (+0.22 g) were most favorable. TGW was strongly affected by the G allele of AX-95210025 at Dharwad (+5.21 g) and the A allele of AX-94633409 at Delhi (+4.29 g). For BM, the G allele of AX-94877518 at Delhi (+0.22 kg/plot) and the A allele of AX-94496657 at Dharwad (+0.19 kg/plot) were key contributors. YLD was polygenic, with the G allele of AX-94877518 showing the strongest effect (+0.07 kg/plot). Heat susceptibility indices highlighted alleles reducing stress effects: HSI_GWPS (T allele of AX-95217431 at Dharwad, -0.35; G allele of AX-94413347 at Pune, -0.36), HSI_TGW (A allele of AX-94734286 at Pune, -0.36), HSI_BM (C allele of AX-94549328, -0.48), and HSI_YLD (A allele of AX-94802270 at Pune, -0.59). These results confirm the polygenic nature of productivity traits and identify stable, high-effect alleles as promising targets for breeding heat-tolerant, high-yielding wheat lines (**Supplementary Table 2**).

## 4 Discussion

The MAGIC population exhibited substantial phenotypic variability for biomass (BM), grain weight per spike (GWPS), thousand grain weight (TGW), and grain yield (YLD) under both timely and late-sown conditions. This wide variation is indicative of diverse genetic backgrounds contributed by the founders and highlights the suitability of MAGIC populations for dissecting complex traits. Heat susceptibility indices (HSIs) also showed broad variation, ranging from tolerant to highly susceptible responses, thereby enabling identification of contrasting lines for stress evaluation. Similar phenotypic ranges for yield and its components under heat stress have been reported by Mason et al. (2010), confirming that genetic diversity within MAGIC populations captures both high productivity and stress-tolerant genotypes.

### 4.1 Biomass and grain traits as key determinants of yield stability and heat stress resilience in the MAGIC population

Correlation analysis revealed significant interrelationships among yield and its component traits under different environmental conditions. Consistent with the findings of Mason et al. (2010) and Correia et al. (2022), biomass stability and grain size emerged as key determinants of heat stress resilience in wheat. A consistently strong positive correlation between grain yield (YLD) and biomass (BM) underscores the pivotal role of biomass accumulation as a primary driver of yield performance. Grain weight per spike (GWPS) and thousand-grain weight (TGW) also exhibited significant positive correlations with yield, reflecting the combined influence of sink capacity (GWPS) and grain size (TGW) in determining final yield (**Figure 2**). In contrast, heat susceptibility index (HSI) traits showed strong negative correlations with productivity-related traits (**Figure 2**), indicating a clear trade-off between heat sensitivity and yield potential.

The principal component analysis (PCA) of the MAGIC wheat population across different sowing conditions and locations revealed clear patterns in the relationships between productivity traits and heat susceptibility indices. Genotypes with higher yield potential generally exhibit lower heat susceptibility, confirming a strong negative correlation between productivity and stress sensitivity (**Figures 3a-f**). In agreement with Zewdu et al. (2024), yield-related traits such as grain weight per spike, biomass yield, grain yield, and thousand-kernel weight were the major contributors to the first principal component (PC1), which explained the largest proportion (42.55%) of phenotypic variation (Ali et al., 2021).

### 4.2 Assessment of population structure and genome-wide linkage disequilibrium decay in the MAGIC population

The identification of distinct subpopulations within the MAGIC panel indicates residual genetic stratification despite multiple generations of intercrossing among founder lines. Such structure likely reflects differential parental genome contributions and selection during line advancement. Accounting for this stratification is essential in downstream association analyses to minimize spurious marker-trait associations and improve the precision of QTL detection (Akond et al., 2021; Cardon and Palmer, 2003; Lou et al., 2026). The moderate extent of genome-wide linkage disequilibrium (3.49 Mb) observed in the population suggests sufficient historical recombination to enhance mapping resolution while still maintaining detectable marker–trait linkages (**Figure 4**). This LD pattern is characteristic of multi-parent populations, where repeated intermating events break long haplotypes into smaller segments. In comparison, Pang et al., 2020 reported a relatively larger LD block size of 4.4 Mb, indicating a slower rate of LD decay than observed in the current MAGIC population. Together, the presence of manageable population structure and relatively rapid LD decay underscores the suitability of the MAGIC population for high-resolution genetic dissection of complex traits such as heat tolerance and yield stability (Huang et al., 2015; Jiang and Xu, 2024; Bag et al., 2026).

### 4.3 SNP associated with yield-related traits under stress conditions

A total of 114 significant marker-trait associations (MTAs) were identified for biomass (BM), grain weight per spike (GWPS), thousand grain weight (TGW), grain yield (YLD), and heat susceptibility indices (HSI) in the MAGIC wheat population evaluated under timely sown irrigated condition (TSIR) and late sown irrigated condition (LSIR)/heat stress condition across multiple locations including Dharwad, Pune, and Delhi (**Table 4**, **Supplementary Table 1**). For biomass (BM), significant MTAs were detected across chromosomes 1A, 1B, 1D, 3A, 4B, 5A, 5D, 6A, 7A, and 7B/D, under both TSIR and LSIR conditions, with major loci such as AX-94942005 (6A), AX-94496657 (6A) and AX-95210025 (5A) explaining up to 13.7% of phenotypic variance explained (PVE), indicating strong genetic control of biomass accumulation under heat stress. These results are consistent with the findings of Molero et al. (2019), SNPs associated with biomass identified at various growth stages explaining up to 12.53% PVE, and Mathew et al. (2019), MTAs associated with biomass reported across chromosomes 1B, 2B, 3A, 4D, and 7A. For grain weight per spike (GWPS), significant MTAs were predominantly clustered on chromosome 2B. Major SNPs including AX-94529210, AX-94948666, AX-95204353, and AX-95118494 explained up to 15.3% PVE, highlighting a key genomic hotspot on chromosome 2B governing spike sink strength under thermal stress (**Table 4**, **Supplementary Table 1**). These findings are in agreement with previous studies reporting QTLs for grain weight per spike on chromosomes 1A, 2B, 3A, 3B, and 6B (Marcotuli et al., 2017; Simmonds et al., 2014; Heidari et al., 2011; Katz et al., 2022).

Regarding thousand grain weight (TGW), significant SNP AX-95210025 (5A) showed a strong and stable association, explaining up to 13.3% PVE, underscoring its major role in regulating grain size and weight. These results corroborate previous studies that reported TGW-associated QTLs on 5A (Wang et al., 2017; Liu et al., 2018; Ahmed et al., 2020; Hu et al., 2016). For grain yield (YLD), significant MTAs were detected across chromosomes 1A, 2B, 2D, 4B, 5A, 6A, 6D and 7D, with major loci such as AX-94942005 (6A), AX-94394403 (2B), AX-95118494 (2B) and AX-94877518 (7D) explaining up to 13.6%, suggesting their importance in maintaining yield stability (**Table 4**, **Supplementary Table 1**). Previous studies by Sukumaran et al. (2015) and Wang et al. (2017) similarly reported yield-associated SNPs on 5A and 6A, while Li et al. (2019) identified 120 loci using SNP and haplotype-based GWAS, including functionally relevant genes contributing to yield formation.

Heat susceptibility indices further revealed the genetic basis of stress resilience, with several MTAs for HSI_BM mapped on chromosomes 3A, 4A, 4D, 5A, 6A, 6B, and 7A, MTAs for HSI_GWPS on chromosomes 1A, 1B, 1D, 2A, 2B, 3A, 3D, 4A, 4B, 5A, 5D, 6D, 7B, and 7D, MTAs for HSI_TGW on chromosomes 1B, 2D, 3D, 5D, 6A, and 7B, and MTAs for HSI_YLD mapped on chromosome 2B, 4B, 5B, 6A, 6B, 7A and 7B. Among these, loci such as AX-94949506 (6A), AX-95249443 (3A), AX-94590438 (2A), AX-94840526 (6B) and AX-94734286 (2D) explained up to 10.7% PVE, indicating moderate to strong contributions to heat tolerance of yield-related traits (**Table 4**, **Supplementary Table 1**). These results align with Mason et al. (2010); Paliwal et al. (2012); Bhusal et al. (2017); Patel et al. (2024)and Awlachew et al. (2016), who identified QTLs for HSI on several corresponding chromosomes.

Several SNPs, including AX-94942005, AX-95210025, AX-95118494, and AX-94734286, were repeatedly detected across multiple traits and environments, highlighting their pleiotropic effects and stability under heat stress. Major MTAs identified for BM, GWPS, TGW, YLD, and their respective HSIs across environments, particularly the stable loci on chromosomes 5A, 2B, 2D and 6A under heat stress conditions. Most of the significant MTAs identified for GWPS were concentrated on chromosome 2B, indicating a potential genomic hotspot for grain weight per spike under heat stress conditions.

### 4.4 Allelic effects of significant SNPs on yield-related traits in the MAGIC population

Allelic effect analysis in the MAGIC population revealed that multiple significant SNPs contributed additively to key productivity and heat-susceptibility traits, including grain weight per spike (GWPS), thousand grain weight (TGW), biomass (BM), grain yield (YLD), and heat susceptibility indices (HSI), under both timely-sown (TSIR) and late-sown (LSIR) conditions across Delhi, Dharwad, and Pune (**Supplementary Table 2**). The magnitude and direction of allelic effects varied among loci, reflecting the polygenic control of these traits.

For GWPS, several SNPs showed strong positive allelic effects across environments. Under TSIR at Delhi, the A allele of AX-94529210 exhibited the highest positive effect (+0.39 g), while under LSIR, the C allele of AX-95204353 and the G allele of AX-94430710 increased GWPS by approximately +0.22 g, demonstrating their stability across sowing conditions. At Dharwad (TSIR) and Pune (LSIR), moderate yet consistent effects were recorded for AX-94701164 (+0.14 g) and AX-94428441 (+0.19 g), respectively, supporting the presence of environmentally stable genomic regions enhancing grain weight under stress (**Supplementary Table 2**). These findings align with earlier reports by Li et al. (2020), observed that superior alleles increased GWPS by 48-70% and were strongly correlated with TGW (r, 0.988). For TGW, multiple SNPs across different chromosomes exhibited significant additive effects under both sowing regimes, emphasizing its polygenic nature. Under Delhi TSIR, the A allele of AX-94633409 and the C allele of AX-94610897 increased TGW by +4.29 g and +3.41 g, respectively. At Dharwad, the G allele of AX-95210025 exerted the largest effect (+5.21 g), followed by AX-94960903 (+3.77 g) and AX-94732891 (+2.02 g), while AX-94691563 and AX-94734286 showed moderate but consistent effects (+2.37-2.72 g) under LSIR (**Supplementary Table 2**). These results corroborate the findings of Li et al. (2020) and Yang et al. (2020), favorable alleles at loci such as AX-111600193 (4A), AX-109860828 (5B), and AX-108838800 (7D) significantly enhanced TGW, with cumulative effects producing up to 39.21 g average kernel weight. In the case of biomass (BM), several loci contributed positively under both timely and late-sown conditions. The G allele of AX-94877518 (Delhi LSIR) increased BM by +0.22 kg plot^-^¹, while the A allele of AX-94496657 (Dharwad TSIR) and the T allele of AX-94484652 (Dharwad LSIR) enhanced BM by +0.19 kg plot^-^¹ and +0.13 kg plot^-^¹, respectively. Similarly, AX-95255439 (C allele) and AX-94541532 (G allele) exhibited consistent moderate effects (+0.09-0.10 kg plot^-^¹) at Pune, suggesting their potential role in maintaining biomass under heat stress. These findings agree with Ain et al. (2015), who demonstrated that favorable alleles exerted cumulative positive effects on TGW and grain yield, explaining up to 32% of yield variation (**Supplementary Table 2**). For yield per plot (YLD), significant allelic effects were detected across locations and conditions, underscoring a complex multi-locus architecture governing yield stability under heat stress. At Delhi TSIR, the G allele of AX-94877518 and the T allele of AX-94889337 increased yield by +0.07 kg plot^-^¹ and +0.05 kg plot^-^¹, respectively. Under LSIR, the G alleles of AX-94394403 and AX-95118494, along with the A allele of AX-94633968, exhibited stable positive effects (+0.05 kg plot^-^¹). At Dharwad TSIR, the C allele of AX-94942005 and G allele of AX-95210025 enhanced yield by +0.06 kg plot^-^¹ and +0.04 kg plot^-^¹, confirming their contribution to yield stability across environments (**Supplementary Table 2**). These results are consistent with Wang et al. (2021), who identified the *Td99211-G* allele (5A) associated with higher TGW and yield, highlighting its breeding potential.

Heat susceptibility indices (HSI) were also influenced by several SNPs, where favorable alleles contributed to reduced sensitivity under stress. In Delhi, the C allele of AX-94527824 and the G allele of AX-95246262 reduced HSI_GWPS by 0.31, while at Dharwad, the T allele of AX-95217431 and C allele of AX-94494989 reduced it by 0.35 and 0.34, respectively. In Pune, the G allele of AX-94498693 and AX-94413347 reduced HSI_GWPS by up to 0.36. Similarly, the A allele of AX-94734286 and the C allele of AX-95199802 reduced HSI_TGW by 0.36 and 0.25, while the C allele of AX-94549328 reduced HSI_BM by 0.48. The A allele of AX-94802270 and AX-94425817 decreased HSI_YLD by 0.59 and 0.32, respectively (**Supplementary Table 2**). These results indicate that specific alleles contribute to thermotolerance by minimizing yield and biomass loss under heat stress.

### 4.5 *In-silico* candidate gene analysis identified complex genetic regulation of productivity under heat stress condition

SNPs associated with biomass, grain weight, yield, and heat stress indices were predominantly located near genes involved in protein phosphorylation, ubiquitin-mediated protein turnover, transcriptional regulation, redox metabolism, and stress-adaptive developmental pathways (**Supplementary Table 3**). SNP AX-95210025 mapped near *TraesCS5A02G388800*, which encodes a non-specific serine/threonine protein kinase involved in the regulation of biomass under abiotic stress conditions (Li et al., 2020), while SNP AX-94942005 (6A) was mapped near *TraesCS6A02G376200/500*, encoding protein kinase domain-containing proteins that mediate stress-responsive signaling pathways sustaining growth under adverse conditions (Ur et al., 2019). Another BM-associated SNP, AX-94818117 (5A)was located near *TraesCS5A02G397200*, encoding a bHLH domain-containing protein involved in biomass regulation under abiotic stress (Wang et al., 2019), and was also positioned close to *TraesCS5A02G396800*, a WRKY transcription factor implicated in biomass accumulation and stress tolerance through transcriptional reprogramming (Yang et al., 2025). Several SNPs for grain weight per spike (GWPS) clustered on chromosome 2B, near genes regulating grain size and filling. SNP AX-94529210 was located near *TraesCS2B02G162000*, encoding a RING-type E3 ubiquitin transferase involved in grain weight regulation (Capron et al., 2012). SNP AX-95204353 mapped close to *TraesCS2B02G193000*, a protein kinase domain-containing gene regulating grain weight through MAPK-mediated developmental signaling (Gasparis and Miłoszewski, 2023). Additionally, SNP AX-95118494 was associated with *TraesCS2B02G186100* and *TraesCS2B02G185800*, encoding a cytochrome P450 protein and Protein kinase domain-containing protein respectively, known to influence grain weight per spike (Gunupuru et al., 2018), while SNP AX-94430710 was positioned near *TraesCS2B02G195400*, an RRM domain-containing protein involved in grain size determination (Yan et al., 2024). For thousand grain weight (TGW), SNP AX-95210025 (5A) was mapped near *TraesCS5A02G388900/389000/389900*, encoding a DUF1618 domain-containing protein reported to influence seed set and grain development, thereby affecting TGW (Luo et al., 2024). SNP AX-94633409 (2B) was associated with *TraesCS2B02G167100*, a GPI-anchored protein regulating kernel endosperm development and TGW (Tian et al., 2023), whereas SNP AX-94734286 (2D) mapped close to *TraesCS2D02G044900*, an NB-ARC domain-containing protein implicated in TGW regulation (Wu et al., 2022). For grain yield (YLD), SNP AX-94877518 (7D) was located near *TraesCS7D02G546500*, encoding an AAA+ ATPase involved in abiotic stress regulation (Wan et al., 2017), and *TraesCS7D02G546300*, a Bowman-Birk serine protease inhibitor family protein associated with seed development and stress tolerance (Xie et al., 2021). The SNP AX-94942005 on chromosome 6A showed association with YLD and was mapped near *TraesCS6A02G376200/500* and *TraesCS6A02G375800/900*, which encode protein kinase domain-containing proteins involved in regulating yield through grain size modulation (Gasparis et al., 2023). Additionally, SNP AX-95118494 (2B) showed pleiotropic association with YLD and was again mapped near the cytochrome P450 gene *TraesCS2B02G186100*, as well as TraesCS2B02G185800, a protein kinase domain-containing gene involved in grain size regulation through MAPK signaling (Gasparis et al., 2023), reinforcing its role in yield determination (Gunupuru et al., 2018).

For heat stress index of biomass (HSI_BM), SNP AX-94949506 (6A) was mapped near *TraesCS6A02G404300/400*, encoding Myb/SANT-like domain-containing transcription factors that regulate biomass production under abiotic stress (Song et al., 2020), and *TraesCS6A02G403700*, an AT-hook motif nuclear-localized protein known to increase biomass by delaying leaf senescence (Lim et al., 2007). For heat stress index of GWPS (HSI_GWPS), SNP AX-94590438 (2A) was associated with *TraesCS2A02G438100*, a protein kinase regulating grain filling and developmental signaling under stress (Krishnappa et al., 2023), and *TraesCS2A02G438000*, a RING-type E3 ubiquitin transferase involved in starch biosynthesis and seed development under heat stress (Parveen et al., 2021). Another HSI_GWPS–associated SNP, AX-95249443 (3A), was located near *TraesCS3A02G492200/400/500/600*, encoding EF-hand domain-containing proteins that play key roles in calcium-mediated heat stress tolerance (Kalaipandian et al., 2019). For heat stress index of TGW (HSI_TGW), SNP AX-94734286 (2D) again mapped near (Cytochrome P450) and *TraesCS2D02G045100* (xyloglucan endotransglucosylase/hydrolase), both involved in TGW regulation and heat stress adaptation, and also close to the NB-ARC domain-containing gene *TraesCS2D02G044900*, indicating its consistent role in TGW stability under stress (Wu et al., 2022), while SNP AX-95219657 (7B) was associated with *TraesCS7B02G063700*, encoding an Fe²^+^/2-oxoglutarate–dependent dioxygenase involved in grain development under abiotic stress (Mostafa et al., 2025). Finally, for heat stress index of yield (HSI_YLD), SNP AX-94802270 (7B) was mapped near *TraesCS7B02G491700/900*, encoding NB-ARC domain-containing proteins implicated in yield regulation under abiotic stress conditions (Gudi et al., 2025).

## 5 Conclusion

The MAGIC population exhibited broad phenotypic variability and strong interrelationships among yield and its component traits, emphasizing the crucial role of biomass accumulation as the primary driver of yield performance under both timely and late-sown irrigated conditions. The allelic and gene expression analyses demonstrated that favorable alleles and upregulated candidate genes contributed additively to improved biomass, grain weight, and yield stability under heat stress. Highly significant and stable SNPs, notably AX-94942005 (6A), AX-95210025 (5A), AX-95118494 (2B), AX-94529210 (2B), AX-94761935 (2B), AX-94877518 (7D) and AX-94734286 (2D), showed strong and pleiotropic associations with biomass, grain weight per spike, thousand grain weight, grain yield, and heat susceptibility indices across environments. These SNPs were linked to biologically relevant candidate genes, including protein kinases WRKY transcription factors RING-type E3 ubiquitin transferases, cytochrome P450 and NB-ARC domain–containing proteins, which collectively regulate growth, grain development, and stress adaptation. The GWAS, allelic effect analysis and candidate gene analysis highlight these loci and genes as key determinants of yield stability under heat stress and valuable targets for marker-assisted breeding and genomic selection models to accelerate genetic gain for yield and heat tolerance, thereby enhancing the development of climate-resilient wheat cultivars.

## Data Availability

The original contributions presented in the study are included in the article/**Supplementary Material**. Further inquiries can be directed to the corresponding authors.
