# Supplementary material for: MAGIC population-based genetic dissection of yield-related traits under heat stress in wheat (Triticum aestivum L.)
Source: Front Plant Sci. 2026 Apr 7;17:1790276. doi: 10.3389/fpls.2026.1790276 (PMC13095679; doi:10.3389/fpls.2026.1790276)
Supplement: Supplementary file 1 [file Table1.docx]

**MAGIC Population-Based Genetic Dissection of Yield-Related Traits Under Heat Stress in Wheat (*Triticum aestivum* L.)**

Ananta Bag^1^, Hari Krishna*^1^, Vinodh Kumar P N^1^, Shiwani Meena^1^, Narayana Bhat Devate^1^, Rahul Meena^1^, Sudhir Kumar^2^, Ravindra Patil^3^, Uday Govinda Reddy^4^, Amit Kumar Singh^5^, Badal Singh^6^, Neelu Jain^1^, Pradeep Kumar Singh^1^, Gyanendra Pratap Singh*^1^

^1^Division of Genetics, Indian Agricultural Research Institute, New Delhi, India

^2^Division of Plant Physiology, Indian Agricultural Research Institute, New Delhi, India

^3^Genetics and Plant Breeding Group, Agharkar Research Institute, Pune, India

^4^Department of Genetics and Plant Breeding, University of Agricultural Sciences, Dharwad, India

^5^Division of Genomic Resources, ICAR-National Bureau of Plant Genetic Resources, New Delhi, India

^6^Division of Germplasm Evaluation, ICAR-National Bureau of Plant Genetic Resources, New Delhi, India

*Corresponding authors: [harikrishna.agri@gmail.com](mailto:harikrishna.agri@gmail.com) , gyanendrapsingh@hotmail.com

**Supplementary Table 1.** Significant marker-trait associations (MTAs) identified at LOD score > 3 for yield-related traits and their corresponding heat susceptibility indices under TSIR and LSIR conditions across different environments.

| **Traits** | **Environment** | **SNP** | **Chr.** | **Pos** | **P.value** | **R.Squared** | **-log(p)** |
| --- | --- | --- | --- | --- | --- | --- | --- |
| BM | LS_DHAR | AX-94484652 | 4B | 660.65 | 0.0001 | 8.48 | 3.93 |
| BM | LS_DHAR | AX-94818117 | 5A | 591.46 | 0.0003 | 4.18 | 3.57 |
| BM | LS_DL | AX-94877518 | 7D | 633.06 | 0.0003 | 9.41 | 3.52 |
| BM | LS_DL | AX-94987465 | 3A | 744.06 | 0.0001 | 3.58 | 3.9 |
| BM | LS_PUNE | AX-94818117 | 5A | 591.46 | 1E-05 | 7.68 | 4.98 |
| BM | LS_PUNE | AX-94512268 | 5A | 591.49 | 0.0002 | 7.12 | 3.71 |
| BM | LS_PUNE | AX-95230460 | 5A | 592.52 | 0.0001 | 6.82 | 3.91 |
| BM | LS_PUNE | AX-94920868 | 5D | 8.0338 | 4E-05 | 6.32 | 4.37 |
| BM | LS_PUNE | AX-95210025 | 5A | 585.41 | 5E-05 | 5.66 | 4.32 |
| BM | LS_PUNE | AX-94980357 | 5A | 585.07 | 0.0003 | 5.15 | 3.6 |
| BM | LS_PUNE | AX-94403832 | 5A | 588.74 | 0.0001 | 4.93 | 3.97 |
| BM | TS_DHAR | AX-94496657 | 6A | 404.7 | 3E-06 | 13.7 | 5.48 |
| BM | TS_DHAR | AX-94942005 | 6A | 598.18 | 3E-09 | 9.25 | 8.49 |
| BM | TS_DHAR | AX-95210025 | 5A | 585.41 | 1E-06 | 7.83 | 5.83 |
| BM | TS_DHAR | AX-95214723 | 7B | 436.19 | 0.0002 | 3.82 | 3.82 |
| BM | TS_DL | AX-95202637 | 1A | 99.886 | 0.0001 | 4.35 | 3.92 |
| BM | TS_PUNE | AX-95255439 | 1D | 460.79 | 0.0002 | 6.6 | 3.62 |
| BM | TS_PUNE | AX-94541532 | 1B | 632.53 | 2E-05 | 5.6 | 4.67 |
| BM | TS_PUNE | AX-95205723 | 7A | 85.836 | 1E-04 | 5.45 | 4.01 |
| BM | TS_PUNE | AX-94568900 | 3A | 27.533 | 0.0001 | 5.21 | 3.97 |
| GWPS | LS_DHAR | AX-94561239 | 7A | 13.627 | 0.0001 | 5.77 | 3.94 |
| GWPS | LS_DL | AX-95204353 | 2B | 168.61 | 7E-05 | 7.78 | 4.18 |
| GWPS | LS_DL | AX-94430710 | 2B | 172.98 | 0.0003 | 5.86 | 3.56 |
| GWPS | TS_DHAR | AX-94701164 | 6A | 611.49 | 0.0003 | 10.6 | 3.53 |
| GWPS | TS_DL | AX-94529210 | 2B | 135 | 3E-05 | 15.3 | 4.6 |
| GWPS | TS_DL | AX-95104040 | 2B | 182.01 | 0.0001 | 12.2 | 3.83 |
| GWPS | TS_DL | AX-95204353 | 2B | 168.61 | 7E-05 | 11 | 4.18 |
| GWPS | TS_DL | AX-94761935 | 2B | 161.42 | 0.0001 | 10.4 | 3.95 |
| GWPS | TS_DL | AX-94430710 | 2B | 172.98 | 7E-05 | 10.1 | 4.17 |
| GWPS | TS_DL | AX-95118494 | 2B | 161.15 | 7E-05 | 10 | 4.18 |
| GWPS | TS_DL | AX-94622328 | 2B | 162.93 | 8E-05 | 9.91 | 4.09 |
| GWPS | TS_DL | AX-94948666 | 2B | 412.66 | 4E-05 | 9.53 | 4.41 |
| GWPS | TS_DL | AX-94450544 | 2B | 163.71 | 0.0002 | 8.67 | 3.67 |
| GWPS | TS_DL | AX-94478188 | 2A | 141.9 | 0.0002 | 6.39 | 3.68 |
| GWPS | TS_DL | AX-95130119 | 6B | 710.1 | 0.0002 | 6.29 | 3.61 |
| GWPS | TS_DL | AX-95259848 | 6D | 465.21 | 0.0002 | 5.85 | 3.67 |
| GWPS | TS_PUNE | AX-94428441 | 6B | 20.999 | 4E-07 | 7.02 | 6.43 |
| TGW | LS_DHAR | AX-94691563 | 5A | 585.49 | 0.0001 | 4.44 | 3.86 |
| TGW | LS_PUNE | AX-94734286 | 2D | 16.358 | 0.0002 | 6.57 | 3.78 |
| TGW | TS_DHAR | AX-95210025 | 5A | 585.41 | 3E-13 | 13.3 | 12.5 |
| TGW | TS_DHAR | AX-94960903 | 6D | 4.6986 | 0.0001 | 5.72 | 3.94 |
| TGW | TS_DHAR | AX-94853678 | 2D | 615.06 | 0.0002 | 3 | 3.78 |
| TGW | TS_DL | AX-94633409 | 2B | 139.92 | 0.0002 | 6.72 | 3.68 |
| TGW | TS_DL | AX-94610897 | 2D | 58.664 | 0.0002 | 4.5 | 3.69 |
| TGW | TS_PUNE | AX-95254042 | 2A | 715.3 | 0.0002 | 5.4 | 3.64 |
| TGW | TS_PUNE | AX-94902381 | 7B | 1.0276 | 0.0002 | 5.2 | 3.69 |
| TGW | TS_PUNE | AX-94732891 | 2A | 715.41 | 0.0002 | 4.52 | 3.67 |
| YLD | LS_DHAR | AX-94818117 | 5A | 591.46 | 0.0002 | 6.81 | 3.67 |
| YLD | LS_DHAR | AX-94426780 | 5A | 595.54 | 5E-05 | 6.77 | 4.29 |
| YLD | LS_DHAR | AX-95230460 | 5A | 592.52 | 0.0003 | 6.57 | 3.52 |
| YLD | LS_DHAR | AX-95210025 | 5A | 585.41 | 0.0002 | 6.29 | 3.71 |
| YLD | LS_DHAR | AX-94484652 | 4B | 660.65 | 0.0003 | 6.13 | 3.52 |
| YLD | LS_DHAR | AX-94512268 | 5A | 591.49 | 0.0002 | 4.42 | 3.72 |
| YLD | LS_DL | AX-94394403 | 2B | 157.71 | 0.0002 | 11.4 | 3.82 |
| YLD | LS_DL | AX-94633968 | 2D | 107.4 | 0.0001 | 10.7 | 3.87 |
| YLD | LS_DL | AX-95118494 | 2B | 161.15 | 6E-05 | 10.6 | 4.25 |
| YLD | LS_DL | AX-94622328 | 2B | 162.93 | 0.0001 | 10.5 | 3.96 |
| YLD | LS_DL | AX-94761935 | 2B | 161.42 | 0.0002 | 10 | 3.78 |
| YLD | LS_DL | AX-94755547 | 2B | 164.42 | 0.0002 | 5.27 | 3.77 |
| YLD | LS_DL | AX-94503076 | 1A | 473.79 | 0.0003 | 4.09 | 3.53 |
| YLD | LS_DL | AX-94766218 | 2B | 164.11 | 0.0002 | 3.97 | 3.61 |
| YLD | LS_PUNE | AX-94571927 | 2B | 3.5043 | 0.0003 | 3.98 | 3.51 |
| YLD | LS_PUNE | AX-94514951 | 2B | 2.67 | 0.0001 | 3.74 | 3.91 |
| YLD | LS_PUNE | AX-94775808 | 2B | 5.6016 | 0.0002 | 3.74 | 3.71 |
| YLD | TS_DHAR | AX-94942005 | 6A | 598.18 | 4E-12 | 11.5 | 11.4 |
| YLD | TS_DHAR | AX-95210025 | 5A | 585.41 | 3E-07 | 8.08 | 6.48 |
| YLD | TS_DHAR | AX-94737812 | 2B | 141.85 | 4E-05 | 8.02 | 4.4 |
| YLD | TS_DHAR | AX-94626799 | 2B | 141.88 | 4E-05 | 8.02 | 4.4 |
| YLD | TS_DL | AX-94877518 | 7D | 633.06 | 0.0001 | 13.6 | 3.99 |
| YLD | TS_DL | AX-94889337 | 6D | 458.51 | 0.0001 | 5.82 | 3.97 |
| YLD | TS_DL | AX-94582979 | 1A | 571.18 | 0.0002 | 4.23 | 3.81 |
| YLD | TS_DL | AX-94474833 | 1A | 591.75 | 0.0001 | 3.97 | 3.99 |
| YLD | TS_PUNE | AX-94426780 | 5A | 595.54 | 0.0002 | 5.64 | 3.78 |
| HSI_BM | DHAR | AX-95236410 | 3A | 2.1977 | 0.0006 | 5.8 | 3.21 |
| HSI_BM | DHAR | AX-94549328 | 6B | 32.33 | 0.0003 | 3.82 | 3.46 |
| HSI_BM | DHAR | AX-94540236 | 4D | 230.61 | 0.001 | 3.79 | 3 |
| HSI_BM | DL | AX-94949506 | 6A | 610.93 | 0.0003 | 6.27 | 3.55 |
| HSI_BM | DL | AX-94987465 | 3A | 744.06 | 0.0001 | 4.94 | 3.88 |
| HSI_BM | DL | AX-95188728 | 7A | 366.94 | 0.0003 | 4 | 3.54 |
| HSI_BM | PUNE | AX-94922900 | 3A | 620.76 | 0.0002 | 4.53 | 3.71 |
| HSI_BM | PUNE | AX-94515020 | 5A | 493.86 | 0.0003 | 4.35 | 3.53 |
| HSI_BM | PUNE | AX-94911547 | 5A | 592.3 | 0.0004 | 4.34 | 3.39 |
| HSI_BM | PUNE | AX-94959684 | 3D | 614.37 | 0.0006 | 4.27 | 3.25 |
| HSI_BM | PUNE | AX-95126527 | 7A | 4.9381 | 0.0004 | 3.6 | 3.4 |
| HSI_BM | PUNE | AX-95195143 | 4A | 734.96 | 0.0008 | 3.17 | 3.12 |
| HSI_GWPS | DHAR | AX-94590438 | 2A | 689.71 | 0.0005 | 8.11 | 3.27 |
| HSI_GWPS | DHAR | AX-95249443 | 3A | 719.3 | 5E-05 | 6.42 | 4.33 |
| HSI_GWPS | DHAR | AX-94885021 | 3D | 590.21 | 0.0002 | 5.34 | 3.79 |
| HSI_GWPS | DHAR | AX-95239994 | 1D | 109.98 | 6E-06 | 5.32 | 5.23 |
| HSI_GWPS | DHAR | AX-94414057 | 2B | 648.92 | 0.0005 | 5.27 | 3.29 |
| HSI_GWPS | DHAR | AX-94494989 | 4A | 740.45 | 0.0005 | 4.74 | 3.3 |
| HSI_GWPS | DHAR | AX-94525583 | 3A | 717.65 | 0.0003 | 4.61 | 3.54 |
| HSI_GWPS | DHAR | AX-95217431 | 4A | 733.49 | 0.0008 | 4.44 | 3.07 |
| HSI_GWPS | DHAR | AX-94921260 | 6D | 418.89 | 0.0005 | 4.42 | 3.28 |
| HSI_GWPS | DHAR | AX-94888072 | 5A | 442.35 | 0.0009 | 3.6 | 3.07 |
| HSI_GWPS | DHAR | AX-94701190 | 3A | 719.76 | 0.0001 | 3.59 | 3.92 |
| HSI_GWPS | DHAR | AX-94663713 | 5D | 399.29 | 0.0007 | 3.51 | 3.15 |
| HSI_GWPS | DHAR | AX-94976763 | 3D | 609.94 | 4E-06 | 3.42 | 5.35 |
| HSI_GWPS | DHAR | AX-94534520 | 6D | 6.1343 | 0.0003 | 3.3 | 3.53 |
| HSI_GWPS | DL | AX-94433739 | 1B | 557.66 | 0.0001 | 4.02 | 3.88 |
| HSI_GWPS | DL | AX-95657292 | 1A | 508.32 | 6E-05 | 3.75 | 4.25 |
| HSI_GWPS | DL | AX-94718117 | 1B | 564.74 | 0.0003 | 3.72 | 3.52 |
| HSI_GWPS | DL | AX-94763810 | 5D | 435.25 | 0.0009 | 3.47 | 3.05 |
| HSI_GWPS | DL | AX-95109822 | 1D | 420.55 | 0.0006 | 3.46 | 3.21 |
| HSI_GWPS | DL | AX-94527824 | 1D | 412.08 | 0.0005 | 3.34 | 3.3 |
| HSI_GWPS | DL | AX-95246262 | 1A | 508.23 | 0.0001 | 3.32 | 3.99 |
| HSI_GWPS | DL | AX-94618537 | 1A | 510.7 | 0.0002 | 3.29 | 3.61 |
| HSI_GWPS | DL | AX-95071361 | 1A | 508.52 | 0.0003 | 3.21 | 3.47 |
| HSI_GWPS | DL | AX-94926956 | 4B | 621.33 | 0.0009 | 3.21 | 3.04 |
| HSI_GWPS | DL | AX-94586921 | 1A | 514.48 | 0.0004 | 3.14 | 3.44 |
| HSI_GWPS | PUNE | AX-94845319 | 7D | 252.86 | 0.0009 | 4.9 | 3.04 |
| HSI_GWPS | PUNE | AX-95024666 | 5D | 550.61 | 0.0004 | 4.57 | 3.4 |
| HSI_GWPS | PUNE | AX-94413347 | 7B | 224.7 | 0.001 | 3.85 | 3.01 |
| HSI_GWPS | PUNE | AX-95126743 | 7B | 356.83 | 0.0009 | 3.71 | 3.04 |
| HSI_GWPS | PUNE | AX-94498693 | 7B | 210.31 | 0.0008 | 3.69 | 3.09 |
| HSI_GWPS | PUNE | AX-94442813 | 7B | 231.3 | 0.0008 | 3.54 | 3.1 |
| HSI_GWPS | PUNE | AX-94457751 | 7B | 236.76 | 0.001 | 3.43 | 3 |
| HSI_GWPS | PUNE | AX-94503379 | 7B | 457.38 | 0.0006 | 3.28 | 3.19 |
| HSI_TGW | DHAR | AX-95219657 | 7B | 69.355 | 0.0008 | 6.28 | 3.1 |
| HSI_TGW | DHAR | AX-94645037 | 3D | 603.61 | 0.0009 | 4.6 | 3.05 |
| HSI_TGW | DHAR | AX-94475996 | 1B | 48.467 | 0.0002 | 4.22 | 3.66 |
| HSI_TGW | DHAR | AX-94710293 | 5D | 188.83 | 0.0007 | 3.87 | 3.17 |
| HSI_TGW | DL | AX-94795971 | 6A | 600.82 | 0.0006 | 3.91 | 3.21 |
| HSI_TGW | PUNE | AX-94734286 | 2D | 16.358 | 5E-05 | 10.7 | 4.32 |
| HSI_TGW | PUNE | AX-95199802 | 1B | 686.93 | 0.0009 | 3.11 | 3.03 |
| HSI_YLD | DHAR | AX-95658523 | 6A | 599.05 | 0.0002 | 5.2 | 3.62 |
| HSI_YLD | DL | AX-94840526 | 6B | 159.42 | 0.0002 | 8.82 | 3.71 |
| HSI_YLD | DL | AX-94785550 | 4B | 665.81 | 0.0002 | 4.04 | 3.71 |
| HSI_YLD | DL | AX-94991915 | 3B | 807.36 | 0.001 | 3.26 | 3.01 |
| HSI_YLD | PUNE | AX-94802270 | 7B | 749.41 | 0.0004 | 6.03 | 3.42 |
| HSI_YLD | PUNE | AX-94425817 | 7A | 734.47 | 0.0006 | 5.53 | 3.25 |
| HSI_YLD | PUNE | AX-94875764 | 7B | 746.52 | 0.0002 | 5 | 3.78 |
| HSI_YLD | PUNE | AX-94571927 | 2B | 3.5043 | 0.0004 | 4.96 | 3.39 |
| HSI_YLD | PUNE | AX-95186387 | 5B | 10.169 | 0.0001 | 3.91 | 3.91 |

GWPS-Grain weight per spike; TGW-Thousand-grain weight; BM-Biomass; YLD-Grain yield; HSI_GWPS, HSI_TGW, HSI_BM, HSI_YLD – Heat susceptibility indices for respective traits; TS – Timely sown; LS = Late Sown Irrigated (LSIR); TS = Timely Sown Irrigated (TSIR); DL = Delhi; DHAR = Dharwad; PUNE = Pune.

**Supplementary Table 2.** Allelic effects of significant SNPs for GWPS, TGW, BM, YLD, and their corresponding heat susceptibility indices in the MAGIC Population across environments and sowing conditions.

| **Trait** | **Conditions** | **SNPs** | **Alleles** | **Mean ± SD** | **Allele effect** | **t stat** | **t critical** | **P value** | **Sign** |
| --- | --- | --- | --- | --- | --- | --- | --- | --- | --- |
| GWPS | TS_DL | AX-94529210 | T | 2.44±0.31 | 0.39 | 7.21 | 1.66 | 7.00E-11 | *** |
|  |  |  | C | 2.05±0.34 |  |  |  |  |  |
| GWPS | TS_DL | AX-95104040 | T | 2.46±0.3 | 0.37 | 7.07 | 1.66 | 7.00E-11 | *** |
|  |  |  | C | 2.08±0.35 |  |  |  |  |  |
| GWPS | TS_DL | AX-95204353 | C | 2.49±0.28 | 0.29 | 6.4 | 1.65 | 5.00E-10 | *** |
|  |  |  | G | 2.2±0.37 |  |  |  |  |  |
| GWPS | TS_DL | AX-94761935 | C | 2.52±0.27 | 0.28 | 6.36 | 1.65 | 8.00E-10 | *** |
|  |  |  | G | 2.24±0.36 |  |  |  |  |  |
| GWPS | TS_DL | AX-94430710 | G | 2.52±0.27 | 0.3 | 6.63 | 1.65 | 1.00E-10 | *** |
|  |  |  | A | 2.22±0.37 |  |  |  |  |  |
| GWPS | TS_DL | AX-95118494 | G | 2.52±0.27 | 0.28 | 6.43 | 1.65 | 6.00E-10 | *** |
|  |  |  | A | 2.24±0.36 |  |  |  |  |  |
| GWPS | TS_DL | AX-94622328 | G | 2.52±0.27 | 0.29 | 6.41 | 1.65 | 7.00E-10 | *** |
|  |  |  | T | 2.24±0.36 |  |  |  |  |  |
| GWPS | TS_DL | AX-94948666 | G | 2.51±0.29 | 0.33 | 6.22 | 1.65 | 2.00E-09 | *** |
|  |  |  | A | 2.18±0.38 |  |  |  |  |  |
| GWPS | TS_DL | AX-94450544 | C | 2.5±0.27 | 0.27 | 5.97 | 1.65 | 6.00E-09 | *** |
|  |  |  | T | 2.23±0.36 |  |  |  |  |  |
| GWPS | LS_DL | AX-95204353 | C | 2±0.29 | 0.22 | 5.5 | 1.65 | 6.00E-08 | *** |
|  |  |  | G | 1.77±0.29 |  |  |  |  |  |
| GWPS | LS_DL | AX-94430710 | G | 2.01±0.29 | 0.22 | 5.22 | 1.65 | 2.00E-07 | *** |
|  |  |  | A | 1.79±0.29 |  |  |  |  |  |
| GWPS | TS_DHAR | AX-94701164 | C | 2.23±0.14 | 0.14 | 4.27 | 1.7 | 9.00E-05 | *** |
|  |  |  | T | 2.09±0.14 |  |  |  |  |  |
| GWPS | LS_PUNE | AX-94428441 | T | 1.93±0.23 | 0.19 | 2.89 | 1.71 | 0.0039 | ** |
|  |  |  | C | 1.75±0.28 |  |  |  |  |  |
| TGW | TS_DL | AX-94633409 | A | 50±4.22 | 4.29 | 4.11 | 1.71 | 0.0002 | *** |
|  |  |  | G | 45.7±4.25 |  |  |  |  |  |
| TGW | TS_DL | AX-94610897 | C | 49.5±4.26 | 3.41 | 3.77 | 1.68 | 0.0003 | *** |
|  |  |  | T | 46.1±4.73 |  |  |  |  |  |
| TGW | TS_DHAR | AX-95210025 | G | 53.4±4.36 | 5.21 | 6.72 | 1.66 | 3.00E-10 | *** |
|  |  |  | A | 48.1±5.9 |  |  |  |  |  |
| TGW | TS_DHAR | AX-94960903 | A | 54.2±4.7 | 3.77 | 4.63 | 1.66 | 5.00E-06 | *** |
|  |  |  | G | 50.4±5.69 |  |  |  |  |  |
| TGW | LS_DHAR | AX-94691563 | A | 36.9±3.51 | 2.37 | 2.53 | 1.7 | 0.0084 | ** |
|  |  |  | C | 34.5±4.54 |  |  |  |  |  |
| TGW | TS_PUNE | AX-95254042 | A | 44±2.86 | 1.59 | 4.03 | 1.65 | 4.00E-05 | *** |
|  |  |  | G | 42.4±2.73 |  |  |  |  |  |
| TGW | TS_PUNE | AX-94902381 | T | 43.8±2.76 | 1.36 | 3.22 | 1.66 | 0.0008 | *** |
|  |  |  | G | 42.5±2.6 |  |  |  |  |  |
| TGW | TS_PUNE | AX-94732891 | A | 43.6±2.93 | 2.02 | 3.35 | 1.7 | 0.0011 | ** |
|  |  |  | T | 41.6±2.67 |  |  |  |  |  |
| TGW | LS_PUNE | AX-94734286 | A | 38±3.74 | 2.72 | 3.95 | 1.66 | 8.00E-05 | *** |
|  |  |  | G | 35.3±4.05 |  |  |  |  |  |
| BM | TS_DL | AX-95202637 | T | 1.27±0.18 | 0.1 | 1.24 | 2.13 | 0.1416 | ns |
|  |  |  | C | 1.17±0.18 |  |  |  |  |  |
| BM | LS_DL | AX-94877518 | G | 0.8±0.19 | 0.22 | 4.42 | 1.75 | 0.0002 | *** |
|  |  |  | A | 0.58±0.18 |  |  |  |  |  |
| BM | TS_DHAR | AX-94496657 | A | 1.06±0.18 | 0.19 | 5.61 | 1.68 | 7.00E-07 | *** |
|  |  |  | C | 0.87±0.16 |  |  |  |  |  |
| BM | LS_DHAR | AX-94484652 | T | 0.81±0.19 | 0.13 | 3.48 | 1.68 | 0.0005 | *** |
|  |  |  | C | 0.68±0.21 |  |  |  |  |  |
| BM | LS_DHAR | AX-94818117 | C | 0.82±0.19 | 0.09 | 2.98 | 1.66 | 0.0017 | ** |
|  |  |  | T | 0.74±0.21 |  |  |  |  |  |
| BM | TS_PUNE | AX-95255439 | C | 1.54±0.15 | 0.09 | 3.96 | 1.65 | 6.00E-05 | *** |
|  |  |  | T | 1.45±0.15 |  |  |  |  |  |
| BM | TS_PUNE | AX-94541532 | G | 1.54±0.15 | 0.1 | 4.94 | 1.65 | 1.00E-06 | *** |
|  |  |  | A | 1.44±0.14 |  |  |  |  |  |
| BM | TS_PUNE | AX-95205723 | G | 1.61±0.16 | 0.13 | 3.23 | 1.73 | 0.0022 | ** |
|  |  |  | C | 1.48±0.15 |  |  |  |  |  |
| BM | LS_PUNE | AX-94818117 | T | 1.24±0.17 | 0.11 | 4.79 | 1.66 | 2.00E-06 | *** |
|  |  |  | C | 1.14±0.13 |  |  |  |  |  |
| BM | LS_PUNE | AX-94512268 | G | 1.26±0.17 | 0.1 | 3.65 | 1.67 | 0.0003 | *** |
|  |  |  | A | 1.15±0.15 |  |  |  |  |  |
| BM | LS_PUNE | AX-95230460 | C | 1.29±0.17 | 0.12 | 3.46 | 1.7 | 0.0008 | *** |
|  |  |  | G | 1.16±0.15 |  |  |  |  |  |
| YLD | TS_DL | AX-94877518 | G | 0.41±0.05 | 0.07 | 3.95 | 1.76 | 0.0007 | *** |
|  |  |  | A | 0.34±0.06 |  |  |  |  |  |
| YLD | TS_DL | AX-94889337 | T | 0.41±0.05 | 0.05 | 4.01 | 1.71 | 0.0003 | *** |
|  |  |  | C | 0.36±0.05 |  |  |  |  |  |
| YLD | LS_DL | AX-94394403 | G | 0.26±0.06 | 0.05 | 5.9 | 1.65 | 1.00E-08 | *** |
|  |  |  | C | 0.21±0.05 |  |  |  |  |  |
| YLD | LS_DL | AX-94633968 | A | 0.26±0.06 | 0.05 | 5.86 | 1.66 | 2.00E-08 | *** |
|  |  |  | C | 0.21±0.06 |  |  |  |  |  |
| YLD | LS_DL | AX-95118494 | G | 0.27±0.06 | 0.05 | 6.19 | 1.66 | 4.00E-09 | *** |
|  |  |  | A | 0.21±0.06 |  |  |  |  |  |
| YLD | LS_DL | AX-94761935 | C | 0.26±0.06 | 0.05 | 6.03 | 1.66 | 7.00E-09 | *** |
|  |  |  | G | 0.21±0.06 |  |  |  |  |  |
| YLD | LS_DL | AX-94755547 | C | 0.25±0.06 | 0.04 | 5.32 | 1.65 | 1.00E-07 | *** |
|  |  |  | T | 0.21±0.06 |  |  |  |  |  |
| YLD | TS_DHAR | AX-94942005 | C | 0.36±0.05 | 0.06 | 7.32 | 1.66 | 2.00E-11 | *** |
|  |  |  | T | 0.3±0.05 |  |  |  |  |  |
| YLD | TS_DHAR | AX-95210025 | G | 0.36±0.06 | 0.04 | 4.47 | 1.65 | 8.00E-06 | *** |
|  |  |  | A | 0.32±0.06 |  |  |  |  |  |
| YLD | TS_DHAR | AX-94737812 | C | 0.36±0.06 | 0.03 | 4.23 | 1.65 | 2.00E-05 | *** |
|  |  |  | T | 0.33±0.06 |  |  |  |  |  |
| YLD | TS_DHAR | AX-94626799 | G | 0.36±0.06 | 0.03 | 4.23 | 1.65 | 2.00E-05 | *** |
|  |  |  | A | 0.33±0.06 |  |  |  |  |  |
| YLD | LS_DHAR | AX-94818117 | C | 0.25±0.04 | 0.02 | 3.24 | 1.66 | 0.0007 | *** |
|  |  |  | T | 0.22±0.05 |  |  |  |  |  |
| YLD | LS_DHAR | AX-94426780 | T | 0.25±0.04 | 0.03 | 2.92 | 1.67 | 0.0024 | ** |
|  |  |  | C | 0.22±0.06 |  |  |  |  |  |
| YLD | LS_DHAR | AX-95230460 | G | 0.24±0.05 | 0.04 | 4.11 | 1.7 | 0.0001 | *** |
|  |  |  | C | 0.2±0.05 |  |  |  |  |  |
| YLD | LS_DHAR | AX-95210025 | G | 0.25±0.04 | 0.03 | 3.72 | 1.66 | 0.0001 | *** |
|  |  |  | A | 0.22±0.05 |  |  |  |  |  |
| YLD | LS_DHAR | AX-94484652 | T | 0.24±0.05 | 0.03 | 3.23 | 1.68 | 0.0011 | ** |
|  |  |  | C | 0.21±0.05 |  |  |  |  |  |
| YLD | TS_PUNE | AX-94426780 | T | 0.49±0.05 | 0.03 | 2.82 | 1.67 | 0.0031 | ** |
|  |  |  | C | 0.46±0.06 |  |  |  |  |  |
| HSI_GWPS | DELHI | AX-94718117 | C | 0.98±0.4 | 0.29 | 4.7 | 1.68 | 1.00E-05 | *** |
|  |  |  | A | 0.69±0.29 |  |  |  |  |  |
| HSI_GWPS | DELHI | AX-94763810 | T | 1.03±0.41 | 0.16 | 2.34 | 1.66 | 0.0104 | * |
|  |  |  | C | 0.87±0.39 |  |  |  |  |  |
| HSI_GWPS | DELHI | AX-94527824 | G | 0.98±0.41 | 0.31 | 3.78 | 1.75 | 0.0008 | *** |
|  |  |  | C | 0.67±0.27 |  |  |  |  |  |
| HSI_GWPS | DELHI | AX-95246262 | T | 0.98±0.4 | 0.31 | 3.82 | 1.75 | 0.0007 | *** |
|  |  |  | G | 0.67±0.27 |  |  |  |  |  |
| HSI_GWPS | DELHI | AX-94618537 | C | 0.98±0.4 | 0.29 | 4.67 | 1.68 | 2.00E-05 | *** |
|  |  |  | G | 0.69±0.28 |  |  |  |  |  |
| HSI_GWPS | DELHI | AX-95071361 | C | 0.98±0.4 | 0.3 | 4.42 | 1.7 | 6.00E-05 | *** |
|  |  |  | T | 0.68±0.29 |  |  |  |  |  |
| HSI_GWPS | DELHI | AX-94926956 | A | 1.12±0.46 | 0.22 | 2.88 | 1.67 | 0.0028 | ** |
|  |  |  | G | 0.91±0.38 |  |  |  |  |  |
| HSI_GWPS | DHAR | AX-95249443 | G | 1.03±0.31 | 0.22 | 4.2 | 1.67 | 4.00E-05 | *** |
|  |  |  | A | 0.81±0.3 |  |  |  |  |  |
| HSI_GWPS | DHAR | AX-95239994 | A | 1.13±0.33 | 0.2 | 3.18 | 1.68 | 0.0013 | ** |
|  |  |  | G | 0.93±0.3 |  |  |  |  |  |
| HSI_GWPS | DHAR | AX-94414057 | G | 1.03±0.32 | 0.21 | 3.63 | 1.67 | 0.0003 | *** |
|  |  |  | C | 0.82±0.29 |  |  |  |  |  |
| HSI_GWPS | DHAR | AX-94494989 | T | 1.31±0.22 | 0.34 | 5.29 | 1.75 | 4.00E-05 | *** |
|  |  |  | C | 0.96±0.31 |  |  |  |  |  |
| HSI_GWPS | DHAR | AX-95217431 | G | 1.31±0.25 | 0.35 | 3.83 | 1.86 | 0.0025 | ** |
|  |  |  | T | 0.96±0.3 |  |  |  |  |  |
| HSI_GWPS | DHAR | AX-94921260 | A | 1.29±0.23 | 0.33 | 3.83 | 1.86 | 0.0025 | ** |
|  |  |  | T | 0.96±0.31 |  |  |  |  |  |
| HSI_GWPS | PUNE | AX-94413347 | C | 1.07±0.57 | 0.34 | 3.45 | 1.67 | 0.0006 | *** |
|  |  |  | G | 0.73±0.52 |  |  |  |  |  |
| HSI_GWPS | PUNE | AX-94498693 | A | 1.07±0.57 | 0.36 | 3.7 | 1.68 | 0.0003 | *** |
|  |  |  | G | 0.71±0.5 |  |  |  |  |  |
| HSI_GWPS | PUNE | AX-94442813 | T | 1.07±0.57 | 0.32 | 3.35 | 1.67 | 0.0007 | *** |
|  |  |  | C | 0.75±0.52 |  |  |  |  |  |
| HSI_GWPS | PUNE | AX-94457751 | T | 1.07±0.57 | 0.32 | 3.28 | 1.67 | 0.0009 | *** |
|  |  |  | G | 0.75±0.52 |  |  |  |  |  |
| HSI_TGW | DHAR | AX-95219657 | A | 1.1±0.28 | 0.18 | 1.73 | 1.86 | 0.0606 | ns |
|  |  |  | G | 0.92±0.25 |  |  |  |  |  |
| HSI_TGW | DHAR | AX-94645037 | G | 1.06±0.22 | 0.15 | 3.2 | 1.68 | 0.0012 | ** |
|  |  |  | A | 0.91±0.23 |  |  |  |  |  |
| HSI_TGW | DHAR | AX-94475996 | C | 1.03±0.24 | 0.1 | 3 | 1.65 | 0.0015 | ** |
|  |  |  | T | 0.93±0.24 |  |  |  |  |  |
| HSI_TGW | DHAR | AX-94710293 | T | 1.03±0.24 | 0.1 | 2.92 | 1.65 | 0.002 | ** |
|  |  |  | C | 0.93±0.26 |  |  |  |  |  |
| HSI_TGW | PUNE | AX-94734286 | G | 1.16±0.54 | 0.36 | 4.1 | 1.66 | 5.00E-05 | *** |
|  |  |  | A | 0.8±0.44 |  |  |  |  |  |
| HSI_TGW | PUNE | AX-95199802 | C | 1.06±0.48 | 0.25 | 3.36 | 1.66 | 0.0005 | *** |
|  |  |  | T | 0.81±0.51 |  |  |  |  |  |
| HSI_BM | DHAR | AX-94549328 | T | 1.46±0.54 | 0.48 | 3.52 | 1.73 | 0.0012 | ** |
|  |  |  | C | 0.99±0.51 |  |  |  |  |  |
| HSI_BM | DHAR | AX-94540236 | C | 1.23±0.55 | 0.29 | 3.39 | 1.66 | 0.0005 | *** |
|  |  |  | A | 0.94±0.5 |  |  |  |  |  |
| HSI_BM | PUNE | AX-94515020 | C | 1.13±0.34 | 0.19 | 3.72 | 1.65 | 0.0001 | *** |
|  |  |  | T | 0.93±0.38 |  |  |  |  |  |
| HSI_YLD | DELHI | AX-94840526 | G | 0.99±0.26 | 0.01 | 0.36 | 1.65 | 0.3584 | ns |
|  |  |  | T | 0.98±0.2 |  |  |  |  |  |
| HSI_YLD | DHAR | AX-95658523 | G | 1.04±0.22 | 0.13 | 3.62 | 1.66 | 0.0003 | *** |
|  |  |  | A | 0.91±0.2 |  |  |  |  |  |
| HSI_YLD | PUNE | AX-94802270 | C | 1.5±0.6 | 0.59 | 3.44 | 1.76 | 0.002 | ** |
|  |  |  | A | 0.91±0.55 |  |  |  |  |  |
| HSI_YLD | PUNE | AX-94425817 | G | 1.21±0.66 | 0.32 | 1.94 | 1.73 | 0.034 | * |
|  |  |  | A | 0.89±0.54 |  |  |  |  |  |

SD= standard deviation, t stat = Student’s t-value; t critical = critical t-value at the specified significance level; P value = probability value. Significance levels: * = P < 0.05; ** = P < 0.01; *** = P < 0.001; ns = non-significant. Allele effect of GWPS and TGW are expressed in grams (g), while BM and YLD are expressed in kg plot -1. HSI traits are unitless indices. TS-Timely sown irrigated (TSIR) condition; LS-Late sown irrigated (LSIR) condition; DL-Delhi; DHAR-Dharwad; PUNE-Pune.

**Supplementary Table 3.** Putative candidate genes identified within ±100 kb region of linked SNPs, along with their molecular functions.

| **Traits** | **SNPs** | **Chr. No.** | **Gene ID** | **Proteins** | **Functions** | **References** |
| --- | --- | --- | --- | --- | --- | --- |
| BM | AX-95210025 | 5A | TraesCS5A02G389900 | DUF1618 domain-containing protein | Plant Growth and Development | Lv et al., 2023 |
|  |  | 5A | TraesCS5A02G388800 | non-specific serine/ threonine protein kinase | Regulation of biomass under abiotic stress | Li et al., 2020 |
| BM | AX-94942005 | 6A | TraesCS6A02G376200/500 | Protein kinase domain-containing protein | mediating stress-responsive signaling pathways that sustain growth | Ur et al., 2019 |
| BM | AX-94496657 | 6A | TraesCS6A02G218800 | Bidirectional sugar transporter SWEET | Regulation of biomass production and yield | Wang et al., 2025 |
| BM | AX-94818117 | 5A | TraesCS5A02G396800 | WRKY domain-containing protein | Regulation of biomass and stress tolerance | Yang et al., 2025 |
|  |  | 5A | TraesCS5A02G397200 | BHLH domain-containing protein | Regulation of biomass under abiotic stress | Wang et al., 2019 |
| GWPS | AX-94529210 | 2B | TraesCS2B02G162000 | RING-type E3 ubiquitin transferase | Regulate grain weight by promoting ubiquitin-mediated degradation of proteins. | Capron et al., 2023 |
|  |  | 2B | TraesCS2B02G162400 | Glycosyltransferase | Regulation of grain weight per spike | Gao et al., 2023 |
| GWPS | AX-94948666 | 2B | TraesCS2B02G295900 | RING-type E3 ubiquitin transferase | regulate grain weight under heat stress by modulating starch biosynthesis and seed development | Parveen et al., 2021 |
|  |  | 2B | TraesCS2B02G295800 | RRM domain-containing protein | Regulate grain weight by regulation of grain size | Yan et al., 2024 |
| GWPS | AX-95204353 | 2B | TraesCS2B02G193000 | Protein kinase domain-containing protein | Regulate grain weight by regulation of grain size through MAPK signalling | Gasparis et al., 2023 |
| GWPS | AX-95118494 | 2B | TraesCS2B02G186100 | Cytochrome P450 | Regulation of grain weight per spike | Gunupuru et al., 2018 |
|  |  | 2B | TraesCS2B02G185800 | Protein kinase domain-containing protein | Regulate grain weight by regulation of grain size through MAPK signalling | Gasparis et al., 2023 |
| GWPS | AX-94430710 | 2B | TraesCS2B02G195400 | RRM domain-containing protein | Regulate grain weight by regulation of grain size | Yan et al., 2024 |
| TGW | AX-95210025 | 5A | TraesCS5A02G389100 | Beta-glucosidase | Regulation of TGW by remobilization straw carbon to grain | Wang et al., 2020 |
|  |  | 5A | TraesCS5A02G389900 | DUF1618 domain-containing protein | Influencing 1000-grain weight through its effect on seed set and grain development. | Luo et al., 2024 |
|  |  | 5A | TraesCS5A02G388900/9000 | Beta-glucosidase | Regulation of TGW by remobilization straw carbon to grain | Wang et al., 2020 |
| TGW | AX-94633409 | 2B | TraesCS2B02G167100 | GPI-anchored protein | Regulate TGW bu regulation of kernel endosperm development | Tian et al., 2023 |
| TGW | AX-94734286 | 2D | TraesCS2D02G044900 | NB-ARC domain-containing protein | Regulation of TGW | Wu et al., 2022 |
| YLD | AX-94877518 | 7D | TraesCS7D02G546500 | AAA+ ATPase domain-containing protein | Regulation of abiotic stress | Liu et al., 2015 |
|  |  | 7D | TraesCS7D02G546300 | Bowman-Birk serine protease inhibitors family domain-containing protein | Regulation of abiotic stress, seed development | Xie et al., 2021 |
| YLD | AX-94942005 | 6A | TraesCS6A02G376200/500 | Protein kinase domain-containing protein | Regulate grain weight by regulation of grain size through MAPK signalling | Gasparis et al., 2023 |
|  |  | 6A | TraesCS6A02G375800/900 | Protein kinase domain-containing protein | Regulate yield by regulation of grain size through MAPK signalling | Gasparis et al., 2023 |
| YLD | AX-94394403 | 2B | TraesCS2B02G182800 | NB-ARC domain-containing protein | Regulate grain yield | Wu et al., 2022 |
| YLD | AX-95118494 | 2B | TraesCS2B02G186100 | Cytochrome P450 | Regulation of grain weight | Gunupuru et al., 2018 |
|  |  | 2B | TraesCS2B02G185800 | Protein kinase domain-containing protein | Regulate yield by regulation of grain size through MAPK signalling | Gasparis et al., 2023 |
| HSI_BM | AX-94949506 | 6A | TraesCS6A02G404300/400 | Myb/SANT-like domain-containing protein | Controlling biomass production by regulation of abiotic stress | Song et al., 2020 |
|  |  | 6A | TraesCS6A02G403900 | NB-ARC domain-containing protein | Regulate shoot biomass per plant | Wu |
|  |  | 6A | TraesCS6A02G403700 | AT-hook motif nuclear-localized protein | Increased biomass by reducing leaf senescence | Lim et al., 2007 |
| HSI_GWPS | AX-94590438 | 2A | TraesCS2A02G438500 | F-box domain-containing protein | enhances grain weight per spike under heat stress by maintaining grain size via ubiquitin-mediated regulation. | Li et al., 2024 |
|  |  | 2A | TraesCS2A02G438100 | Protein kinase domain-containing protein | Regulates grain weight by controlling grain filling and developmental signalling pathways in wheat. | Krishnappa et al., 2023 |
|  |  | 2A | TraesCS2A02G438000 | RING-type E3 ubiquitin transferase | regulate grain weight under heat stress by modulating starch biosynthesis and seed development | Parveen et al., 2021 |
| HSI_GWPS | AX-95249443 | 3A | TraesCS3A02G492200/400  /500/600 | EF-hand domain-containing protein | Heat stress tolerance | Kalaipandian et al., 2019 |
| HSI_TGW | AX-94734286 | 2D | TraesCS2D02G044900 | NB-ARC domain-containing protein | Regulation of TGW | Wu et al., 2022 |
|  |  | 2D | TraesCS2D02G044600 | Cytochrome P450 | Regulation of TGW under heat stress | Lv et al., 2025 |
|  |  | 2D | TraesCS2D02G045100 | Xyloglucan endotransglucosylase/hydrolase | Regulate TGW by playing important roles in plant growth, development, and heat stress adaptation. | Bi et al., 2024 |
| HSI_TGW | AX-95219657 | 7B | TraesCS7B02G063700 | Fe2OG dioxygenase domain-containing protein | Regulate development of grains under abiotic stress | Mostafa et al., 2025 |
| HSI_YLD | AX-94802270 | 7B | TraesCS7B02G491700/900 | NB-ARC domain-containing protein | Regulation of yield under abiotic stress | Gudi et al., 2025 |
|  |  | 7B | TraesCS7B02G491300 | FBD domain-containing protein | Abiotic stress tolerance | Wang et al., 2024 |


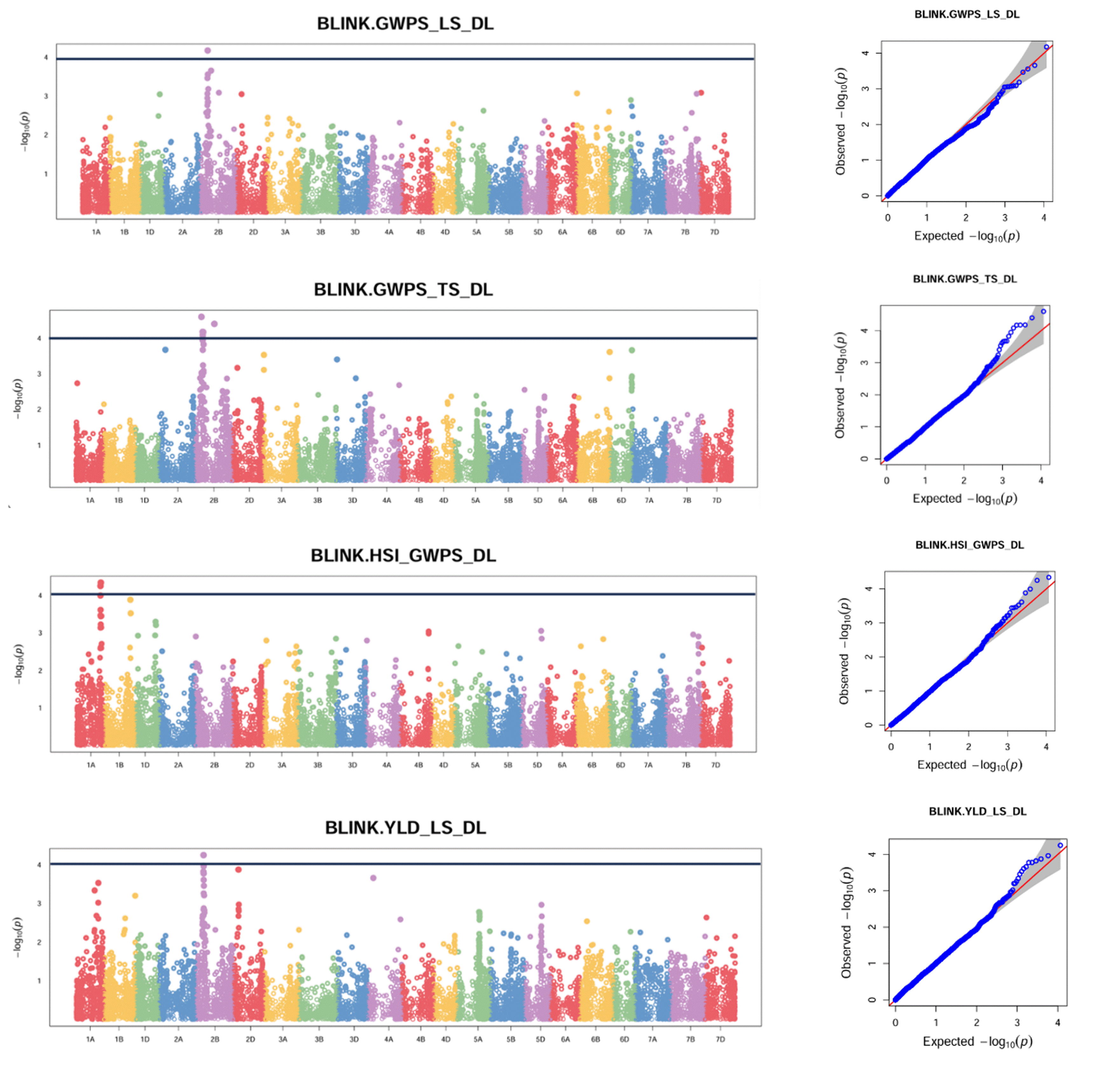


**Supplementary Figure 1** Manhattan plots depicting SNP associations for yield-related traits and their corresponding heat susceptibility indices under TSIR and LSIR conditions at Delhi during the 2024-25 season.


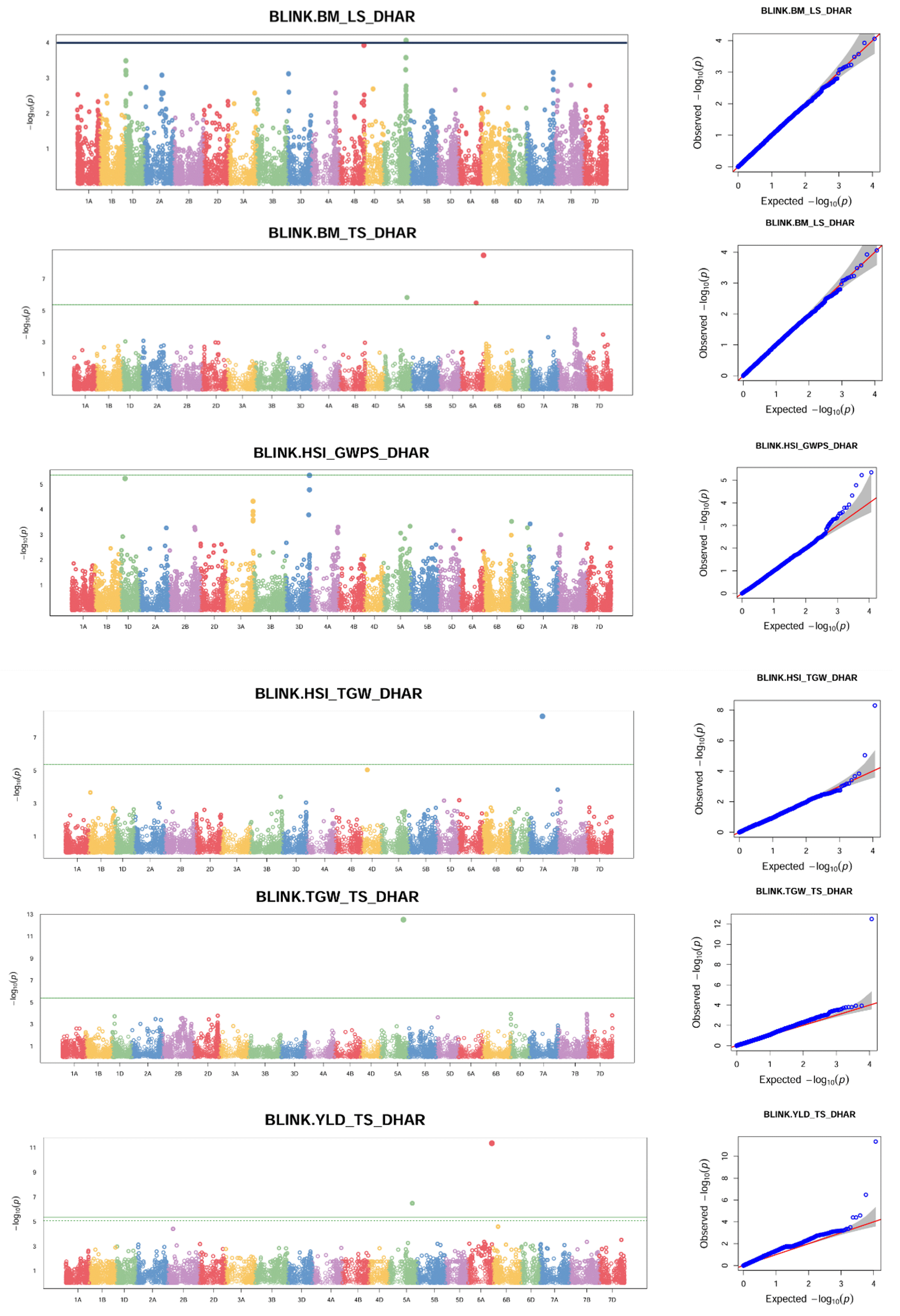


**Supplementary Figure 2** Manhattan plots depicting SNP associations for yield-related traits and their corresponding heat susceptibility indices under TSIR and LSIR conditions at Dharwad during the 2024-25 season.


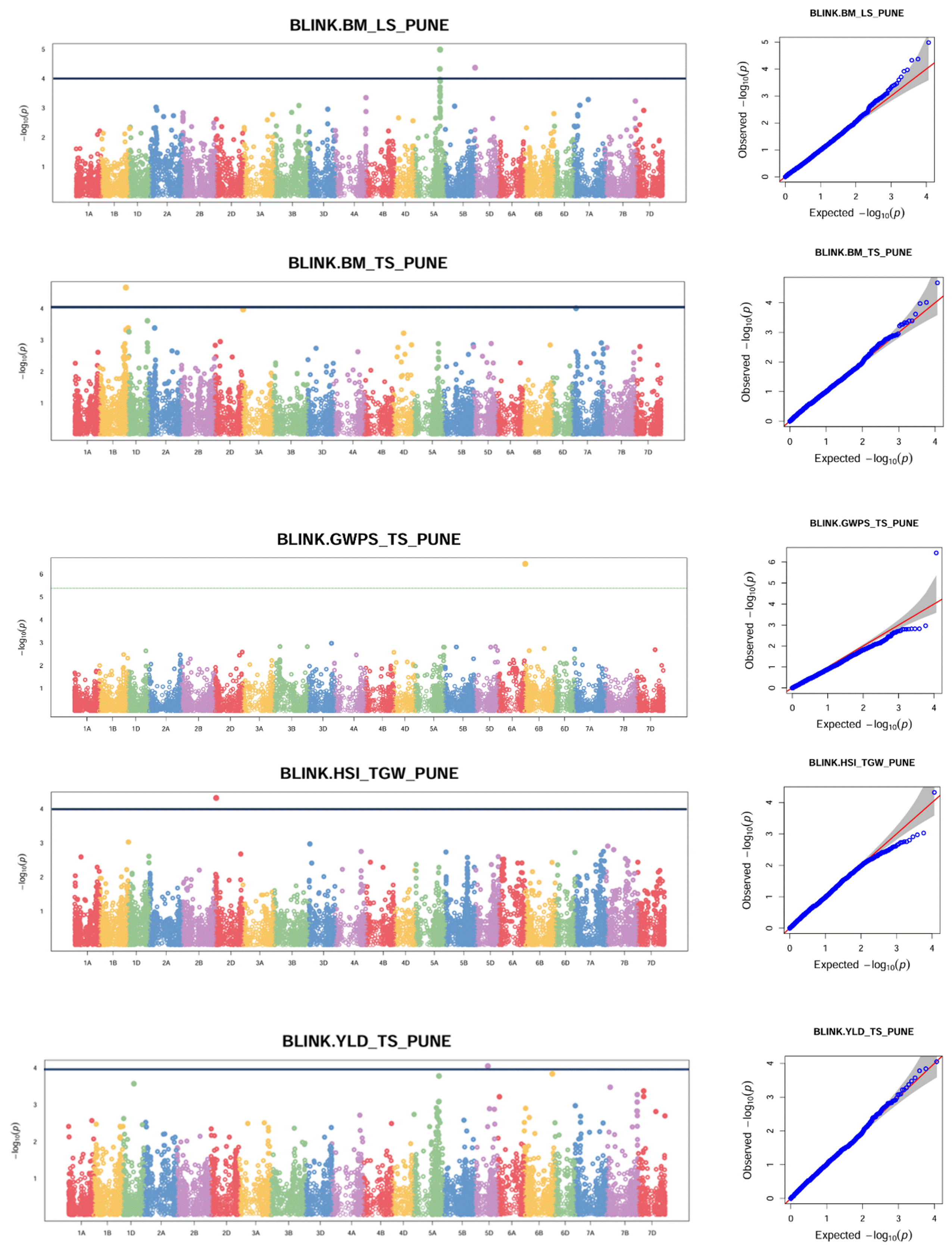


**Supplementary Figure 3.** Manhattan plots depicting SNP associations for yield-related traits and their corresponding heat susceptibility indices under TSIR and LSIR conditions at Pune during the 2024-25 season.

**
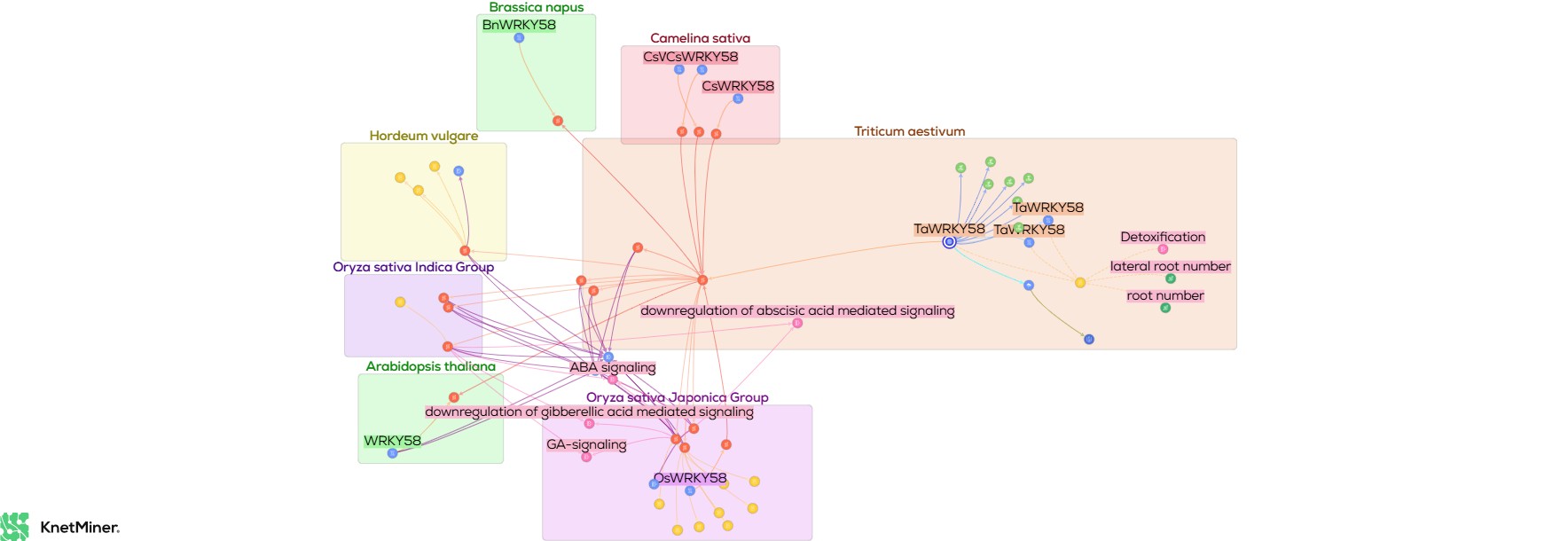
**

**a**

**
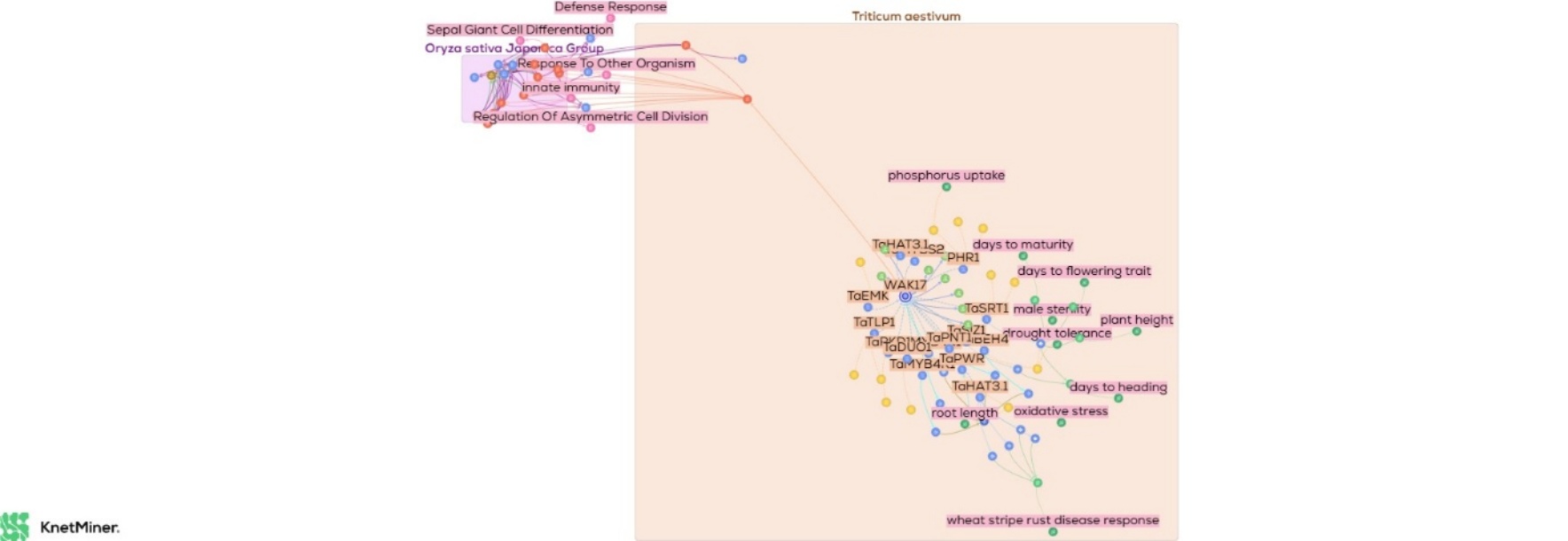
**

**b**

**
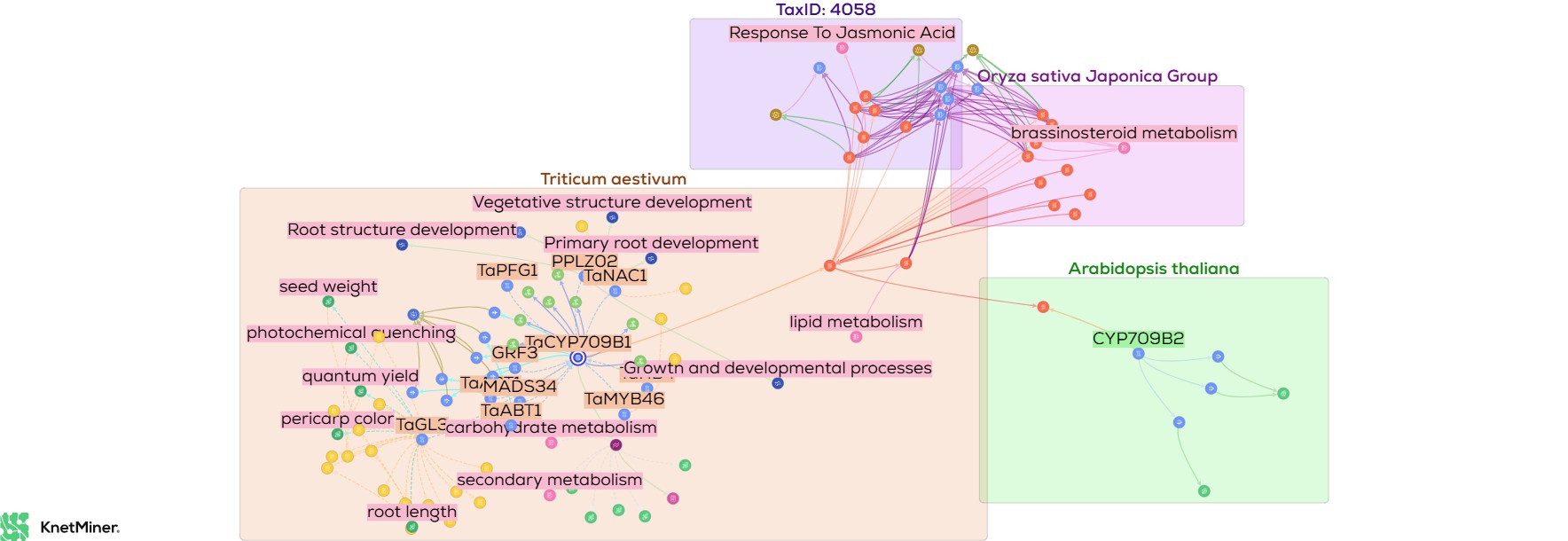
**

**c**

**
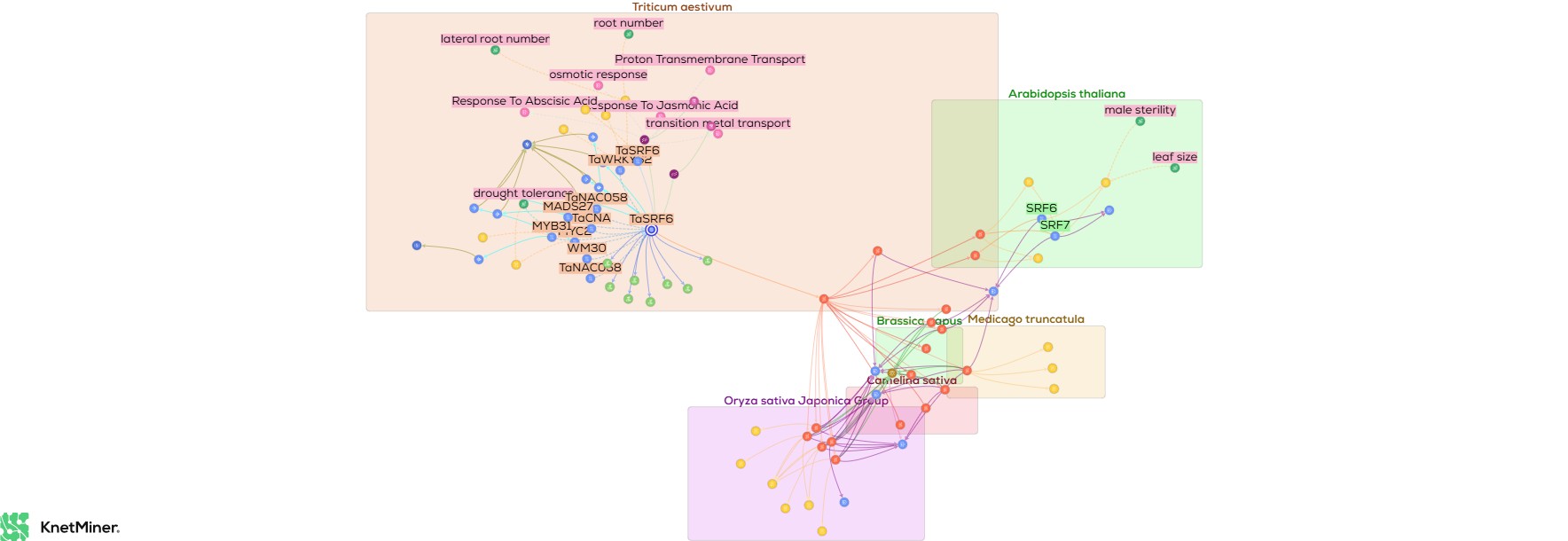
**

**d**

**
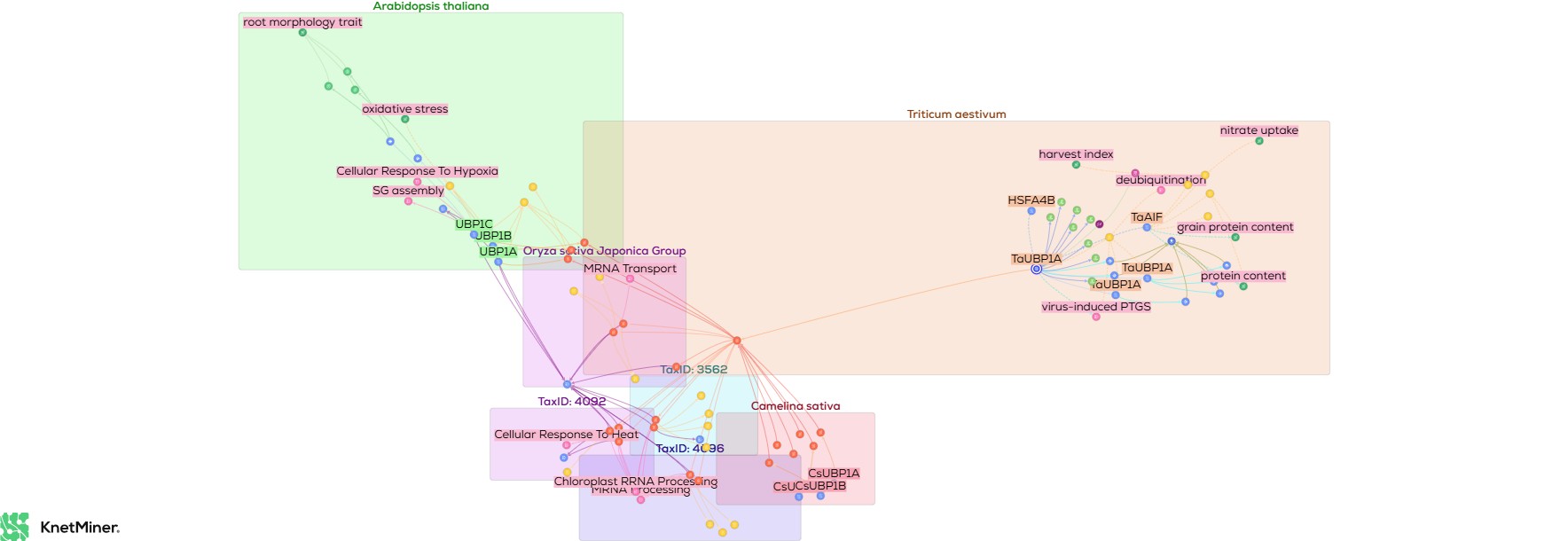
**

**e**

**
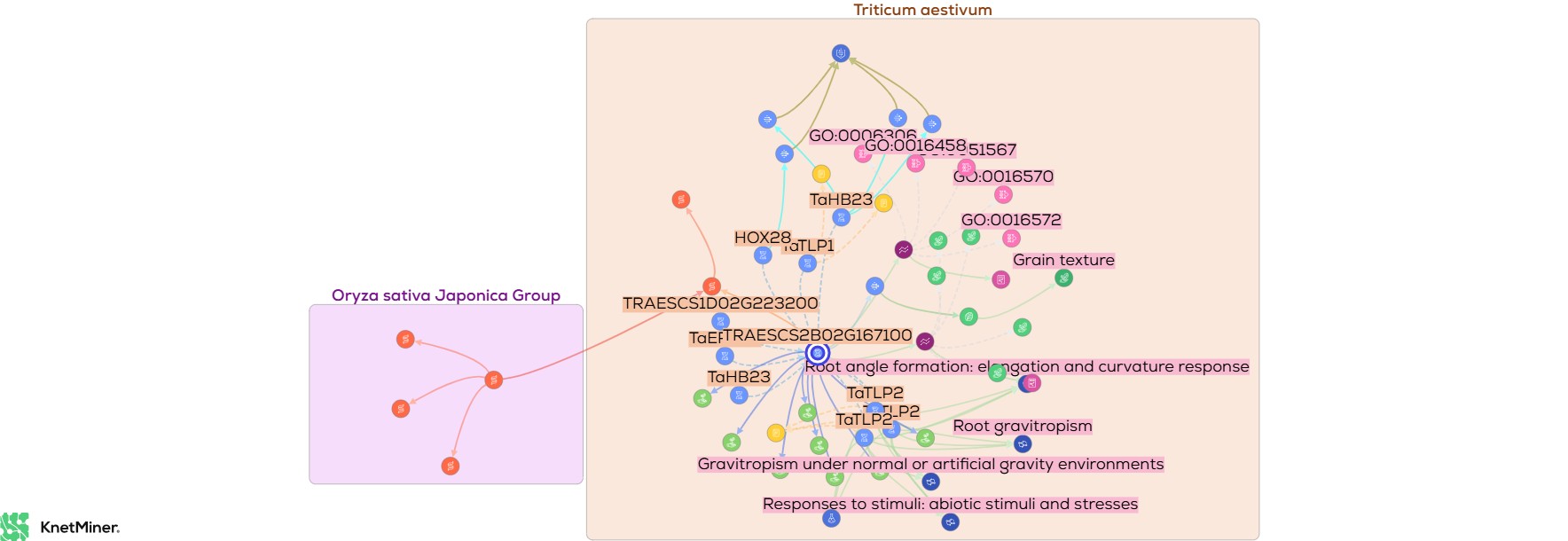
**

**f**

**
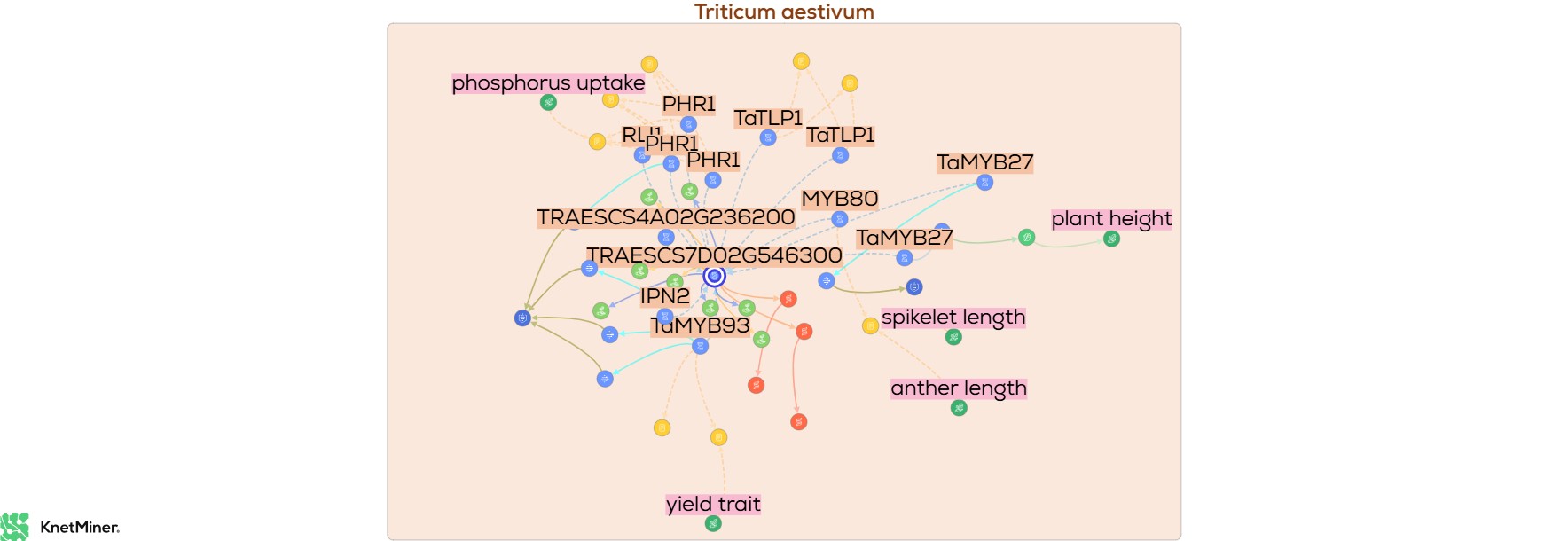
**

**g**

**
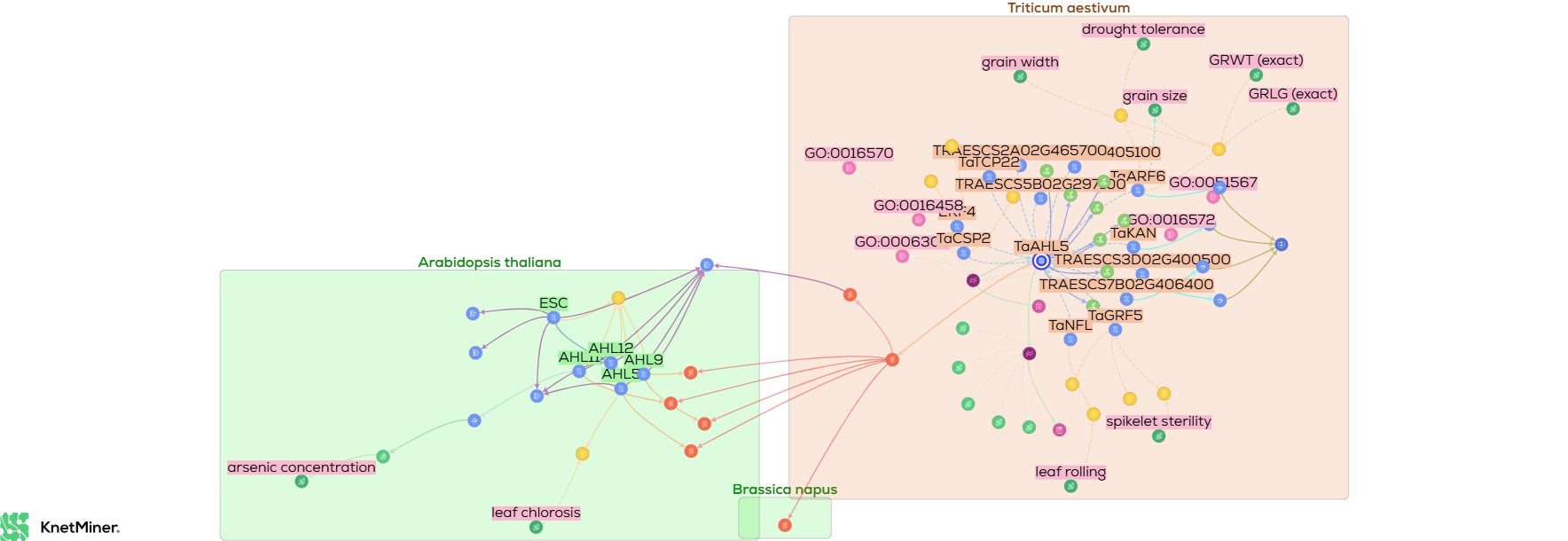
**

**h**

**
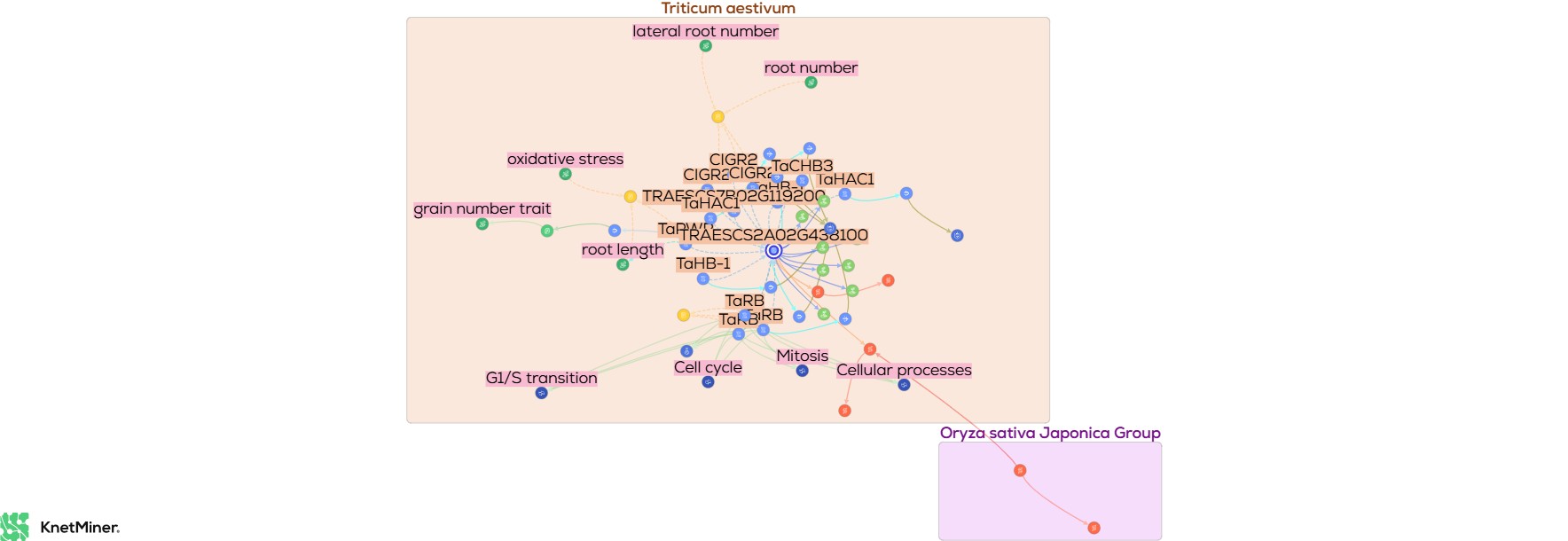
**

**j**

**
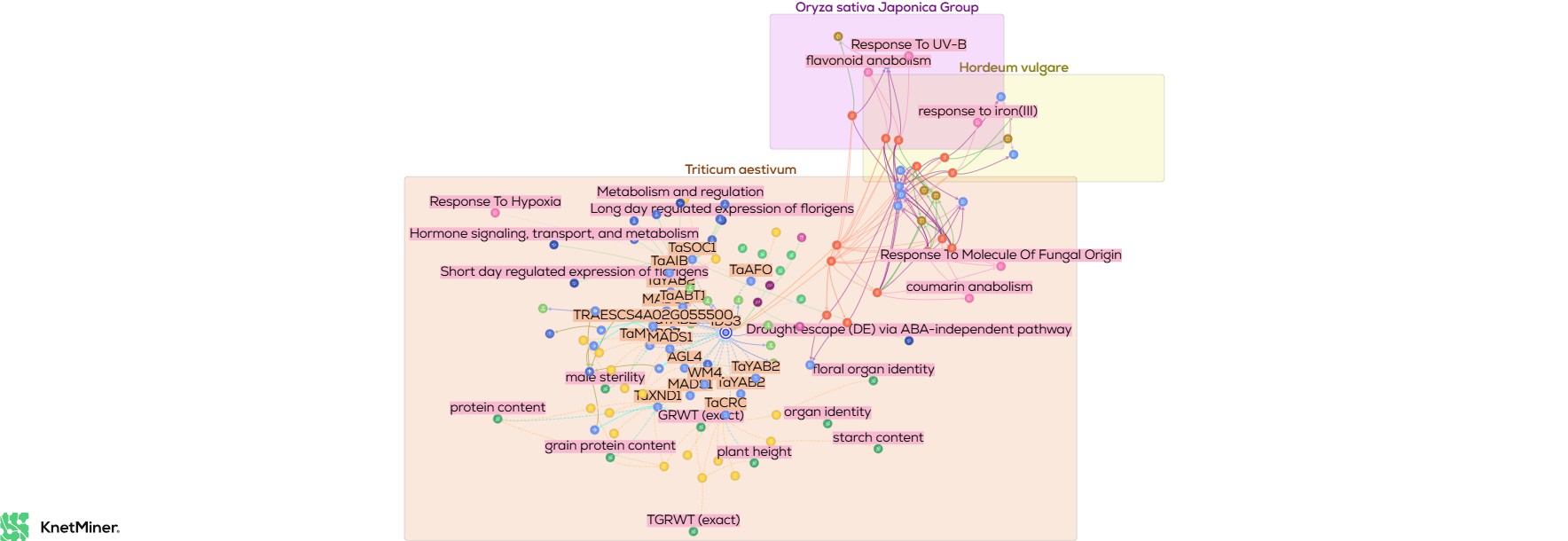
**

**k**

**
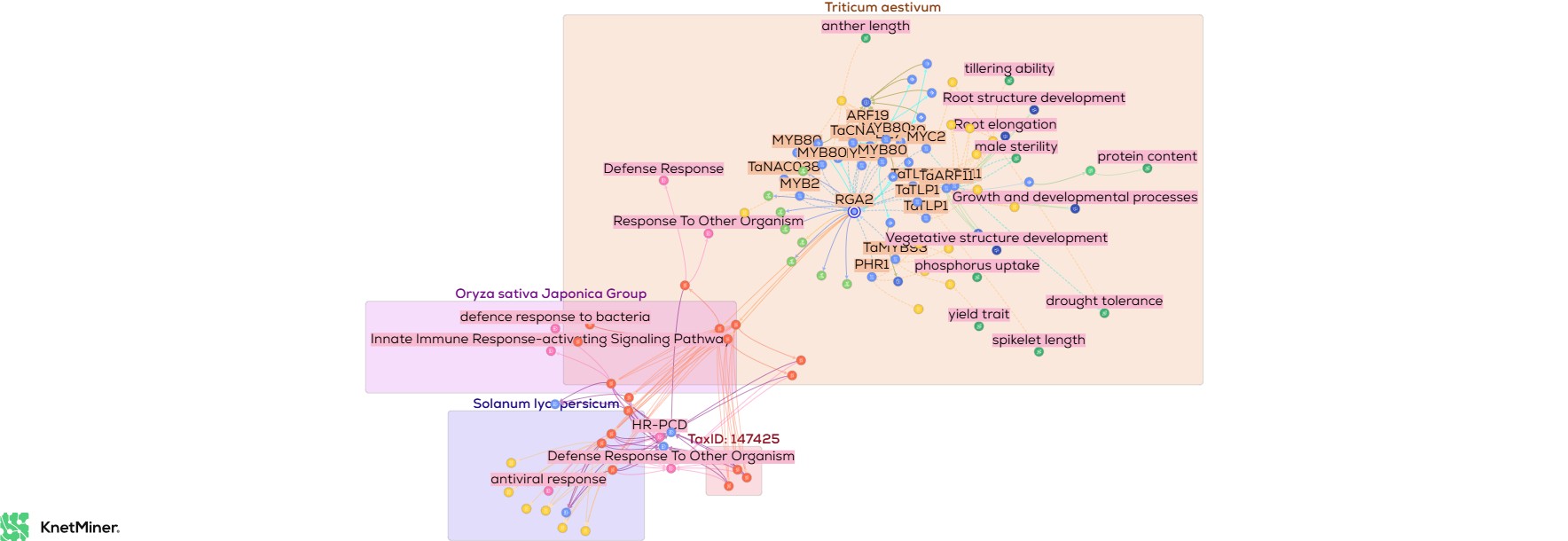
**

**l**

**Supplementary Figure 4** Gene regulatory networks of candidate genes- (**a**) *TraesCS5A02G396800*, (**b**) *TraesCS6A02G376500*, (**c**) *TraesCS2B02G186100*, (**d**) *TraesCS2B02G193000*, (**e**) *TraesCS2B02G195400*, (**f**) *TraesCS2B02G167100*, (**g**) *TraesCS7D02G546300*, (**h**) *TraesCS6A02G403700*, (**i**) *TraesCS2A02G438100*, (**j**) *TraesCS7B02G063700*, (**k**) *TraesCS7B02G063700*, (**l**) *TraesCS7B02G491700*
